# Stereodivergent Synthesis of Aldol Products Using Pseudo-C_2_ Symmetric *N*-benzyl-4-(trifluoromethyl)piperidine-2,6-dione

**DOI:** 10.3390/molecules29215129

**Published:** 2024-10-30

**Authors:** Rina Yada, Tomoko Kawasaki-Takasuka, Takashi Yamazaki

**Affiliations:** Division of Applied Chemistry, Institute of Engineering, Tokyo University of Agriculture and Technology, 2-24-16 Nakamachi, Koganei 184-8588, Japan

**Keywords:** trifluoromethyl, pseudo-C_2_ symmetry, aldol reactions, Michael addition reactions

## Abstract

The present article describes the successful performance of crossed aldol reactions of the CF_3_-containing pseudo-C_2_ symmetric cyclic imide with various aldehydes. The utilization of HMPA as an additive attained the preferential formation of the *anti*-products in good to excellent yields, which contrasts with our previous method without this additive, proceeding to furnish the corresponding *syn*-isomers. The effective participation of ketones and α,β-unsaturated carbonyl compounds in reactions with this imide was also demonstrated to expand the application of this imide.

## 1. Introduction

**Scheme 1:**
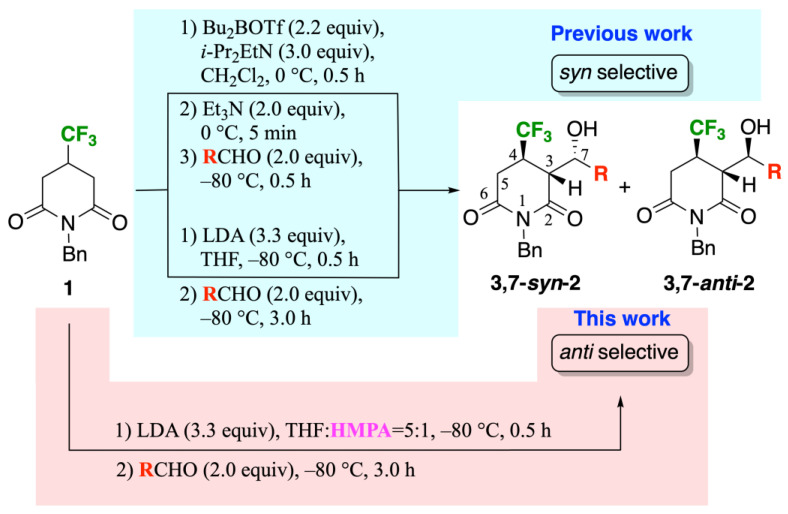
Crossed aldol reactions of **1** with aldehydes.

**Figure 1:**
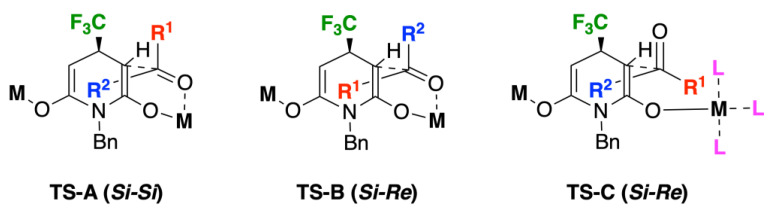
Possible transition state models of the present reactions (**M**: metal, **L**: ligand).

Aldol reactions [1] are well-recognized as convenient and powerful methods for the construction of desired products with the 3-hydroxycarbonyl structure, which was achieved by the nucleophilic attack of enolates on appropriate carbonyl compounds. Their extensive study resulted in the accumulation of a wealth of information to control product stereochemistry [2,3,4,5,6,7,8,9,10,11]. Our ongoing interest in this field has recently culminated in the achievement of the stereoselective aldol reactions starting from *N*-benzyl-4-(trifluoromethyl)piperidine-2,6-dione **1** as the substrate (Scheme 1) [12]. The most significant aspect of this reaction is the utilization of the pseudo-C_2_ symmetric compound [13,14,15,16,17], which facilitates the preparation of products by way of the corresponding bislithium [18,19] or bisboron enolates [4,20] in highly 3,4-*anti*-3,7-*syn* stereoselective fashions. The excellent outcomes obtained by this protocol allowed us to focus on synthesizing the stereoisomeric **3,7-*anti*-2** as the next stage. On the basis of the reaction mechanism that appeared in our previous study, the intermediary bislithium enolates (**M**: Li) were considered to predominantly form the cyclic chelated transition state **TS-A,** and the electrophilic aldehydes (**R^1^**: R, **R^2^**: H) approached from the less-congested enolate *Si* face. The diastereomeric transition state, **TS-B**, is considered to be energetically less stable due to the unfavorable steric interaction between the substrate’s six-membered ring and the substituent **R^1^** (Figure 1), leading to the construction of 3,7-*syn* stereochemistry by way of **TS-A**. Judging from the product stereochemistry, the corresponding bisboron enolate should also favor the same **TS-A**. Then, we envisaged the application of such chelation-breaking reagents **L** like hexamethylphosphoric triamide (HMPA), whose reactions are believed to mainly proceed by way of the different transition state **TS-C**, containing a smaller dipole–dipole interaction between C=O and enolate C-O bonds, to preferentially afford the *anti* products [18,19]. In this article, we would like to disclose our results on the preparation of the 3,7-*anti* diastereoisomers, as well as the reactions of **1** with other electrophiles, like ketones and α,β-unsaturated carbonyl compounds, aiming at the expansion of the utility of substrate **1**.

## 2. Results and Discussion

**Figure 2:**
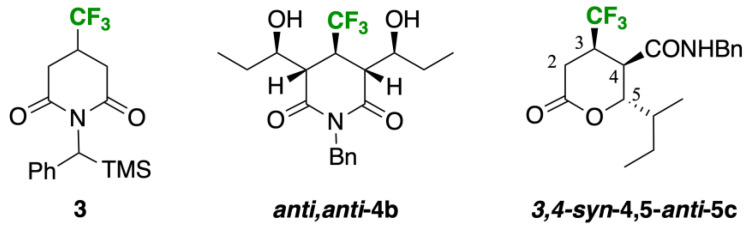
Byproducts of the present reaction.

The present study was initiated to optimize the reaction conditions, whose results are summarized in Table 1. Following our previously established method by the action of a 3.3 equivalent of lithium diisopropylamide (LDA) in THF [12], HMPA was introduced to the resultant bisenolate solution to maintain a tetrahydrofuran (THF)/HMPA volume ratio of 5:1. The brief investigation indicated that the stereoselectivity was sensitive to temperature, the amount of HMPA, and the chelation-breaking reagent used, and the conditions in Entry 2 were found to be the most effective, furnishing product **2a** with 84% 3,7-*anti* selectivity. This result was nicely compared with that obtained without this additive, achieving a 76% 3,7-*syn* selectivity. Using lithium hexamethyldisilazide (LHMDS) as a base instead of LDA was also found to work efficiently, and the employment of a 2.0 equivalent of RCHO provided **3,7-*anti*-2a** in better yield after stirring the mixture for 5 h (Entry 9). It is interesting to note that in the case of LDA, the usage of the same amount of RCHO for lowering the recovery afforded a more complex mixture where the basicity difference of these amides seemed to be influential. Then, a 2.0 equivalent of Me_3_SiCl was added to the bisenolate solution to trap it as the ketene silylacetal, to actually lead to the production of **3** in 37% yield. This result pointed out the possible formation of the trianion intermediate (Figure 2).

In the subsequent stage, the scope and limitations were explored based on the optimal reaction conditions identified in Table 1, utilizing LDA and LHMDS for comparison (Table 2). Both bases recorded the unanimous 3,7-*anti* preference in moderate to good yields. Entries 2 and 3, using propionaldehyde and isobutyraldehyde as the electrophiles, respectively, were the exceptional instances where the particular feature was observed. In the case of the former electrophile with LHMDS as a base, “double aldol product” ***anti*,*anti*-4b** was obtained in 33% yield as a 97:3 stereoisomeric mixture. The structure of the major isomer was deduced from the suggested reaction mechanism in our previous article (Figure 2) [12]. In addition to the expected product **3,7-*anti*-2c**, the lactone **3,4-*syn*-4,5-*anti*-5c** was also produced by the latter aldehyde in 6% (LDA) and 34% (LHMDS) yields, and this lactone formation was likely due to the intramolecular attack of the intermediary alkoxide on the further imide carbonyl group. It is worth noting that these special features were observed only in these two aldehydes, the reason for which is not clear at present.

**Scheme 2:**
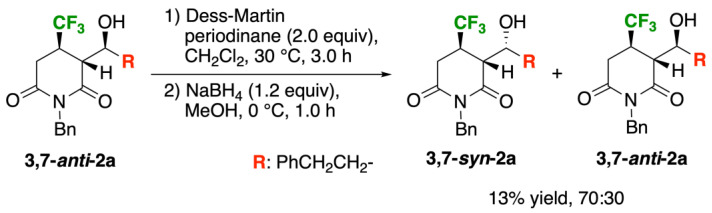
Confirmation of the stereochemistry.

Confirmation of the stereostructure of **3,7-*anti*-2a** was carried out according to our previous report (Scheme 2) [12]. Thus, after the separation from the corresponding 3,7- *syn* isomer by silica-gel column chromatography, **3,7-*anti*-2a** was oxidized by the Dess–Martin reagent, and the obtained ketone was reduced by NaBH_4_ in MeOH without further purification to furnish a mixture of the products **2a** in a ratio of *anti*/*syn*=30:70 (albeit in a low yield). Because the three-dimensional structure of **3,7-*syn*-2a** was already confirmed by the crystallographic analysis [12] and its ^19^F NMR chemical shift was identical to that of the major isomer here, it was clarified that the present method, with the aid of HMPA, actually led to the predominantly stereoselective formation of **3,7-*anti*-2**. 

**Scheme 3:**
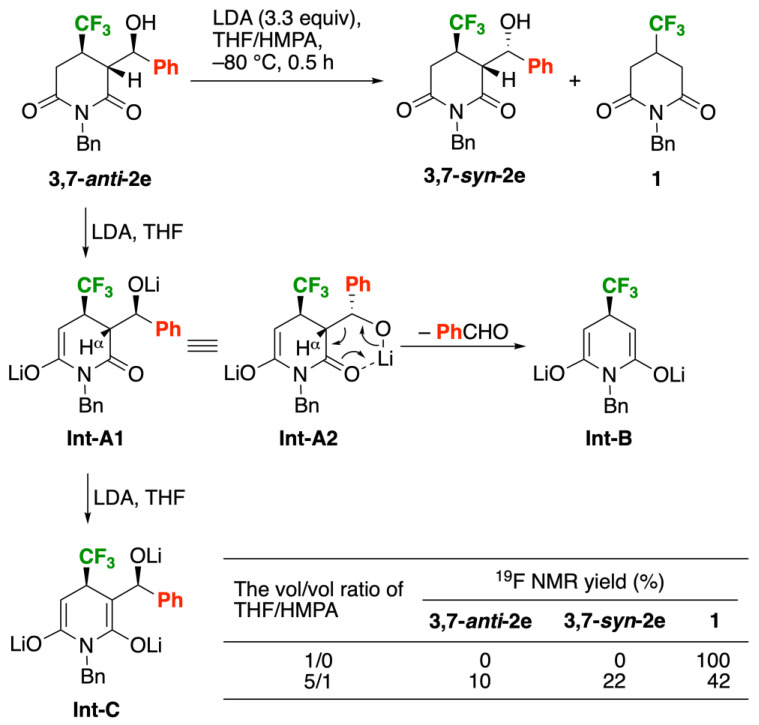
Reaction of ***anti*-2e** with LDA.

To gain more detailed mechanistic insights into the present reaction, chromatographically separated **3,7-*anti*-2e** was added to a solution containing a 3.3 equivalent of LDA at −80 °C for 0.5 h (Scheme 3). When THF was employed as the sole solvent, the total conversion of **3,7-*anti*-2e** to **1** was observed as a result of the retro aldol process. On the other hand, the solvent system of THF:HMPA = 5:1 showed a partial inhibitory effect on the reaction pathway to produce **1** in 42% yield and the isomeric **3,7-*syn*-2e** in 22% yield, with the recovery of **3,7-*anti*-2e** in 10%. This phenomenon can be elucidated as follows. The reaction of a 2 equivalent of LDA with **3,7-*anti*-2e** would afford **Int-A1,** whose rotamer **Int-A2** constructed the more stable intramolecular chelation of Li to the carbonyl oxygen atom, which seemed to facilitate the delivery of PhCHO to produce **Int-B**, leading to **1** upon quenching the reaction. The deprotonation mechanism by LDA involving the chelated six-membered transition state is well-documented [21], and the elimination of H^α^ by a 1 equivalent of LDA in THF would be difficult due to the significant steric congestion. However, this inconvenience would be alleviated by the addition of HMPA, which is considered to change this route to the corresponding non-chelated version. So, the usage of THF as the sole solvent facilitated the reaction to yield **1** by way of **Int-A2**. On the other hand, for the solvent system in the presence of HMPA, because this additive should destroy the chelation, the relatively easier abstraction of H^α^ would make the transformation of **Int-A1** to **Int-C** possible. **Int-C** seems to inhibit the delivery of PhCHO, and a mixture of **3,7-*syn*-** and **3,7-*anti*-2e** was formed by the final protonation.

**Scheme 4:**
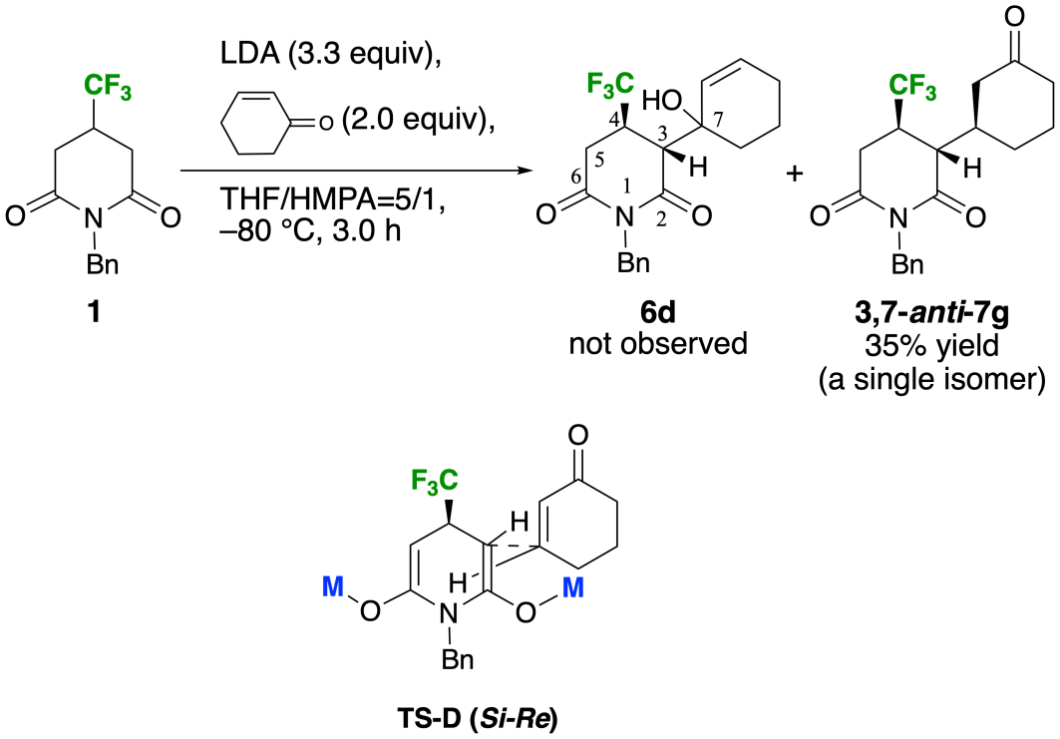
Reaction of **1** with cyclohex-2-en-1-one.

At the next stage, other electrophiles like ketones and α,β-unsaturated carbonyl compounds were selected as the candidates for reactions with imide **1,** whose results are shown in Table 3. Because **6a** was produced only in 17% yield with 75% recovery of **1** when HMPA was used as the additive, THF as the sole solvent was determined as the condition of choice. Reactions of 3-pentanone and 2-butanone with **1** furnished **6a** and **6b**, respectively, only in moderate yields, which would be due to the increase in the steric requirement around the carbonyl group in the electrophile when compared with the cases of aldehydes (Entries 1 and 2). This is actually the case for acetophenone with a bulkier substituent, which failed to yield the addition product **6c** (Entry 3). We also tried Michael addition reactions [22] by use of ethyl acrylate (Entry 4), ethyl (*E*)-but-2-enoate (Entry 5), ethyl (*E*)-3-phenylprop-2-enoate (Entry 6), and phenyl vinyl ketone (Entry 7), all of which nicely reacted with imide **1** to furnish the expected products **7a**–**d** in good to high yields. However, only the recovery of **1** was observed when the α,β-unsaturated amide was used (Entry 8), and this different reactivity from the instances of the structurally similar reactants in Entries 4 to 7 would be understood as the different stability of the intermediary enolates. Thus, it was considered that the less-stable amide enolate intermediate easily returned to a more stable combination of bisenolate from **1** and the α,β-unsaturated amide. Cinnamaldehyde was found to predominantly experience the aldol reaction (Entry 9), which afforded the product **3,7-*syn*-2h** in 15% yield without the detection of the Michael adduct **7f**, which would be a reflection of the high reactivity of the formyl moiety. The reaction of **1** with cyclohex-2-en-1-one was found to be dependent on the solvent employed. Thus, the THF/HMPA solvent system produced the selective synthesis of the 1,4-adduct **3,7-*anti*-7g** in 35% yield as a single stereoisomer with a 35% recovery of **1** (Scheme 4), which was compared with the case when THF was used as a solvent, yielding 1,2-adduct **6d** only in 11% yield with the 85% recovery of **1**.

The relative stereochemistry of the ketone adducts **6,** shown in Table 3, was considered to be 3,4-*anti*-3,7-*syn,* because, in the THF solvent, these reactions seem to follow the same transition state (**TS-A**) in Figure 1 (**R^1^ **> **R^2^**), like the instances of our previous report [12]. On the other hand, it was deduced that the Michael adduct **3,7-*anti*-7g** would be formed by way of **TS-D**. The presence of HMPA should favor the reaction occurring via the non-chelation mechanism, similar to reactions of **1** with aldehydes, as above, and **TS-D** would minimize the unfavorable dipole–dipole interaction as well as the steric hindrance to lead to the formation of the **3,7-*anti*** diastereomer.

## 3. Materials and Methods

**General Information.**^1^H (300.40 MHz), ^13^C (75.45 Hz), and ^19^F (282.65 Hz) NMR spectra were recorded on a AL 300 spectrometer (JEOL Ltd., Tokyo, Japan), and chemical shifts were recorded in parts per million (ppm), downfield from internal tetramethylsilane (Me_4_Si: δ 0.00, for ^1^H and ^13^C) or hexafluorobenzene (C_6_F_6_: δ –163.00 for ^19^F). Data were tabulated in the following order: number of protons, multiplicity (s, singlet; d, doublet; t, triplet; q, quartet; quint, quintet; sex, sextet; m, multiplet; b, broad peak), and coupling constants in Hertz. Infrared (IR) spectra were obtained on a A-302 spectrometer (JASCO Corporation, Tokyo, Japan) and reported in wave numbers (cm^–1^). Elemental analyses were performed using a Series II CHNS/O analyzer (Perkin-Elmer, Shelton, CT, USA). A JMS-700 (JEOL Ltd., Tokyo, Japan) was used for obtaining high-resolution mass spectrometry data in the positive ionization mode.

Most of the reactions where an organic solvent was employed were performed under argon with magnetic stirring using flame-dried glassware. Anhydrous THF, Et_2_O, and CH_2_Cl_2_ were purchased and used without further purification. Unless otherwise noted, materials were obtained from commercial suppliers and were used without further purification. Analytical thin-layer chromatography (TLC) was routinely used for monitoring reactions by generally using a mixture of hexane (Hex) and ethyl acetate (AcOEt) (*v*/*v*). Spherical neutral silica gel (63–210 μm or 40–50 μm) was employed for column chromatography and flush chromatography, respectively.

(2R^*^,3R^*^)-*N*-Benzyl-2-((S^*^)-1-hydroxy-3-phenylpropyl)-3-(trifluoromethyl)-glutarimide, 3,7-*anti*-2a. To a 30 mL three-necked round-bottomed flask, *n*-BuLi (2.6 M: 0.65 mL, 1.7 mmol) was added to a mixture of diisopropylamine (0.25 mL, 1.7 mmol) in THF (4 mL) at −80 °C, which was stirred for 0.5 h at that temperature. To the resultant LDA solution, 0.1356 g of *N*-benzyl-3-(trifluoromethyl)glutarimide **1** (0.500 mmol) [12] was added, and, after 0.5 h, HMPA and THF (1 mL each) were introduced and the whole mixture was stirred for further 0.5 h. Then, 0.12 mL of 3-phenylpropionaldehyde (0.50 mmol) was added and the whole mixture was stirred for 3 h. After quenching this reaction by the addition of 1 M HCl (3.5 mL), the crude material was extracted with Et_2_O three times, and the combined organic phase was dried over anhydrous Na_2_SO_4_. After filtration, the removal of the volatiles furnished the crude materials, which were purified by silica-gel column chromatography (Hex:AcOEt = 5:1) to afford 0.1013 g (0.2499 mmol, 57% yield) of **3,7-*anti*-2a**. For the LHMDS method, hexamethyldisilazane was used instead of diisopropylamine, and the reaction was performed at −80 °C for 5 h to furnish 0.1515 g (0.3737 mmol, 75% yield) of **3,7-*anti*-2a**. **3,7-*anti*-2a**:**3,7-*syn*-2a** = 84:16 (LDA), 89:11 (LHMDS), and Rf = 0.35 (hexane:AcOEt = 3:1)

^1^H NMR: δ 1.80–1.89 (2H, m), 2.58–2.82 (3H, m), 2.88 (1H, d, *J =* 18.0 Hz), 2.91–3.01 (1H, m), 2.95 (1H, s), 3.18 (1H, dd, *J =* 6.9, 17.7 Hz), 4.22 (1H, td, *J =* 4.1, 8.1 Hz), 4.95 (1H, d, *J =* 12.9 Hz), 4.99 (1H, d, *J =* 12.9 Hz), 7.13–7.31 (10H, m). ^13^C NMR: δ 29.5, 32.0, 34.3 (q, *J =* 28.4 Hz), 36.5, 43.3, 45.7, 72.3, 126.4, 126.7 (q, *J =* 278.8 Hz), 127.4, 128.2, 126.2, 128.4, 128.7, 136.5, 140.4, 169.5, 171.8. ^19^F NMR: δ –73.27 (d, *J =* 9.0 Hz), –73.53 (d, *J =* 9.0 Hz: **3,7-*syn*-2a**). IR (CHCl_3_): ν 3485, 3066, 3020, 2946, 1728, 1675, 1391, 1357, 1217, 1187, 1125, 760 cm^–1^. HRMS (FAB+, *m*/*z*): [M+H]^+^ calcd for C_22_H_23_F_3_NO_3_, 405.1630; Found, 405.1611.

(2R^*^,3R^*^)-*N*-Benzyl-2-((S^*^)-1-hydroxypropyl)-3-(trifluoromethyl)glutarimide, 3,7-*anti*-2b. Yield 75% (LDA), 52% (LHMDS). **3,7-*anti*-2b**:**3,7-*syn*-2b** = 94:6 (LDA), 92:8 (LHMDS), Rf = 0.30 (hexane:AcOEt = 3:1). ^1^H NMR: δ 0.97 (3H, t, *J =* 7.4 Hz), 1.59 (2H, dq, *J =* 3.9, 7.5 Hz), 2.23 (1H, s), 2.87–3.01 (3H, m), 3.24 (1H, dd, *J =* 7.2, 18.0 Hz), 4.14 (1H, s), 4.98 (2H, s), 7.22–7.29 (5H, m). ^13^C NMR: δ 9.8, 27.8, 29.5, 34.0 (q, *J =* 28.1 Hz), 43.3, 44.9, 74.2, 126.8 (q, *J =* 279.4 Hz), 127.3, 128.1 128.3, 136.6, 169.6, 172.1. ^19^F NMR: δ −73.19 (d, *J =* 9.0 Hz), −73.59 (d, *J =* 9.0 Hz: **3,7-*syn*-2b**). IR (CHCl_3_): ν 3488, 2968, 2937, 1727, 1671, 1391, 1356, 1225, 1190, 1119, 760 cm^–1^. HRMS (FAB+, *m*/*z*): [M]^+^ calcd for C_16_H_18_F_3_NO_3_, 329.1239; Found, 329.1265.

(2R^*^,3R^*^)-*N*-Benzyl-2-((S^*^)-1-hydroxy-2-methylpropyl)-3-(trifluoromethyl)-glutarimide, 3,7-*anti*-2c. Yield 28% (LDA), 17% (LHMDS). **3,7-*anti*-2c**:**3,7-*syn*-2c** = 61:39 (LDA), >99:<1 (LHMDS), Rf = 0.29 (hexane:AcOEt = 3:1). ^1^H NMR: δ 0.92 (3H, d, *J =* 6.9 Hz), 1.04 (3H, d, *J =* 6.3 Hz), 1.07–1.82 (1H, m), 2.25 (1H, s), 2.89 (1H, d, *J =* 18.6 Hz), 2.90–2.99 (1H, m), 3.09 (1H, s), 3.19 (1H, dd, *J =* 7.5, 18.0 Hz), 3.82–3.86 (1H, m), 4.99 (2H, s), 7.20–7.31 (5H, m). ^13^C NMR: δ 18.4, 18.5, 29.5, 31.2, 34.1 (q, *J =* 27.7 Hz), 43.1, 43.3, 78.9, 126.9 (q, *J =* 279.5 Hz), 127.3, 128.1, 128.3, 136.6, 169.7, 172.3. ^19^F NMR: δ −73.33 (d, *J =* 9.0 Hz), −71.30 (d, *J =* 9.0 Hz: **3,7-*syn*-2c**). IR (CHCl_3_): ν 3505, 2967, 2877, 1728, 1672, 1391, 1356, 1227, 1188, 1119, 759 cm^–1^. HRMS (FAB+, *m*/*z*): [M+H]^+^ calcd for C_17_H_20_F_3_NO_3_, 343.1395; Found, 343.1425.

(2R^*^,3R^*^)-*N*-Benzyl-2-((S^*^)-1-hydroxy-2,2-dimethylpropyl)-3-(trifluoromethyl)-glutarimide, 3,7-*anti*-2d. Yield: 60% (LDA), 26% (LHMDS). **3,7-*anti*-2d**:**3,7-*syn*-2d** = 93:7 (LDA), 77:23 (LHMDS), Rf = 0.30 (hexane:AcOEt = 3:1). ^1^H NMR: δ 1.00 (9H, s), 2.14 (1H, d, *J =* 8.7 Hz), 2.50 (1H, m) 2.86–3.05 (2H, m), 3.31–3.38 (2H, m), 4.53 (1H, d, *J =* 14.4 Hz), 5.04 (1H, d, *J =* 14.1 Hz) 7.25–7.34 (5H, m). ^13^C NMR: δ 25.2, 27.7, 36.9, 41.1 (q, *J =* 27.3 Hz), 42.0, 43.3, 81.3, 126.1 (q, *J =* 280.1 Hz), 127.3, 128.2, 128.5, 136.7, 169.6, 170.1. ^19^F NMR: δ −72.89 (d, *J =* 9.0 Hz), −73.19 (d, *J =* 11.5 Hz: **3,7-*syn*-4d**). IR (CHCl_3_): ν 3475, 3020, 2966, 1731, 1673, 1398, 1360, 1217, 1183, 1128, 754 cm^–1^. HRMS (FAB+, *m*/*z*): [M]^+^ calcd for C_18_H_22_F_3_NO_3_, 357.1552; Found, 357.1560.

(2R^*^,3R^*^)-*N*-Benzyl-2-((S^*^)-1-hydroxy-1-phenylmethyl)-3-(trifluoromethyl)-glutarimide, 3,7-*anti*-2e. Yield 25% (LDA), 32% (LHMDS). **3,7-*anti*-2e**:**3,7-*syn*-2e** = 82:18 (LDA), 88:12 (LHMDS), Rf = 0.35 (hexane:AcOEt = 3:1). ^1^H NMR: δ 2.47 (1H, s), 2.54–2.63 (1H, m), 2.86 (1H, d, *J =* 17.7 Hz), 3.19 (1H, d, *J =* 3.3 Hz), 3.26 (1H, dd, *J =* 7.5, 18.0 Hz), 5.03 (2H, s), 5.55–5.56 (1H, m), 7.14–7.40 (10H, m). ^13^C NMR: δ 29.6, 33.7 (q, *J =* 28.1 Hz), 43.3, 47.1, 75.1, 125.1, 127.0 (q, *J =* 257.4 Hz), 127.3, 128.2, 128.3, 128.5, 129.0, 136.6, 139.7, 169.7, 171.4. ^19^F NMR: δ −73.72 (d, *J =* 11.3 Hz), −73.61 (d, *J =* 11.3 Hz: **3,7-*syn*-2e**). IR (CHCl_3_): ν 3475, 3020, 2966, 1731, 1673, 1398, 1360, 1217, 1183, 1128, 754 cm^–1^. HRMS (FAB+, *m*/*z*): [M]^+^ calcd for C_20_H_18_F_3_NO_3_, 377.1239; Found, 377.1250, [M+H]^+^ calcd for C_20_H_19_F_3_NO_3_, 378.1317; Found, 378.1329.

(2R^*^,3R^*^)-*N*-Benzyl-2-[(S^*^)-1-hydroxy-(4-methoxyphenyl)-methyl)]-3-(trifluoro-methyl)glutarimide, 3,7-*anti*-2f. Yield 18% (LDA), 31% (LHMDS). **3,7-*anti*-2f**: **= >**99:<1 (LDA), 98:2 (LHMDS), Rf = 0.31 (hexane:AcOEt = 3:1). ^1^H NMR: δ 2.30 (1H, d, *J =* 3.9 Hz), 2.57–2.71 (1H, m), 2.87 (1H, d, *J =* 18.0 Hz), 3.15 (1H, s), 3.24 (1H, dd, *J =* 7.8, 18.0 Hz), 3.82 (3H, s), 5.04 (2H, s), 5.50 (1H, t, *J =* 3.9 Hz), 6.90–6.99 (2H, m), 7.23–7.32 (7H, m). ^13^C NMR: δ 29.6, 33.8 (q, *J =* 27.6 Hz), 43.3, 47.3, 55.3, 74.9, 114.4, 126.4, 126.5 (q, *J =* 272.3 Hz), 127.4, 128.3, 131.6, 136.7, 159.6, 169.7, 171.3. ^19^F NMR: δ −73.67 (d, *J =* 9.3 Hz), −73.57 (d, *J =* 11.6 Hz: **3,7-*syn*-2f**). IR (CHCl_3_): ν 3479, 3020, 2958, 2937, 1727, 1674, 1610, 1253, 1221, 1181, 1127, 761 cm^–1^. HRMS (FAB+, *m*/*z*): [M]^+^ calcd for C_21_H_20_F_3_NO_4_, 407.1344; Found, 403.1347.

(2R^*^,3R^*^)-*N*-Benzyl-2-[(S^*^)-1-(4-bromophenyl)-1-hydroxymethyl]-3-(trifluoro-methyl)glutarimide, 3,7-*anti*-2g. Yield 30% (LDA), 46% (LHMDS). **3,7-*anti*-2g**:**3,7-*syn*-2g** = 81:19 (LDA), 88:12 (LHMDS), Rf = 0.37 (hexane:AcOEt = 3:1), White solid, mp.: 123.5 ºC ^1^H NMR: δ 2.51–2.63 (1H, m), 2.71–3.03 (2H, m), 2.87 (1H, d, *J =* 17.7 Hz), 3.14 (1H, s), 3.24 (1H, dd, *J =* 7.5, 18.0 Hz), 5.02 (2H, s), 7.20–7.35 (10H, m). ^13^C NMR: δ 29.6, 33.3 (q, *J =* 28.1 Hz), 43.4, 47.0, 74.5, 122.4, 126.3 (q, *J =* 274.3 Hz), 126.9, 127.4, 128.2, 128.4, 132.2, 136.5, 138.8, 169.5, 171.0. ^19^F NMR: δ −73.72 (d, *J =* 11.3 Hz), −73.59 (d, *J =* 9.0 Hz: **3,7-*syn*-2g**). IR (CHCl_3_): ν 3473, 3065, 3033, 2950, 1728, 1673, 11392, 1356, 1186, 1127, 760, 703 cm^–1^. HRMS (FAB+, *m*/*z*): [M]^+^ calcd for C_20_H_17_BrF_3_NO_3_, 455.0344; Found, 455.0350.

***N*-Benzyl-2-{(*E*)-1-hydroxy-3-phenylprop-2-en-1-yl}-3-(trifluoromethyl)-glutarimide, 3,7-*syn*-2h**. Yield 15%. **3,7-*syn*-2h**:**3,7-*anti*-2h** = 62:38, Rf = 0.31 (hexane:AcOEt = 3:1). ^1^H NMR: **δ** 2.27 (1H, s), 2.88–3.01 (2H, m), 3.10 (1H, dd, *J =* 7.5, 18.0 Hz), 3.23 (1H, s), 4.75–5.78 (1H, m), 4.99 (1H, d, *J =* 13.8 Hz), 5.05 (1H, d, *J =* 13.8 Hz), 6.17 (1H, dd, *J =* 6.9, 15.6 Hz), 6.61 (1H, d, *J =* 15.9 Hz), 7.23–7.35 (10H, m). ^13^C NMR: **δ** 29.7, 36.3 (q, *J =* 29.7 Hz), 43.3, 46.2, 74.1, 126.5 (q, *J =* 276.6 Hz), 126.7, 127.0, 127.5, 128.3, 128.4, 128.5, 128.6, 133.2, 135.2, 136.5, 169.0, 170.0. ^19^F NMR: **δ** −73.27 (d, *J =* 9.0 Hz), −73.38 (d, *J =* 9.6 Hz: **3,7-*anti*-6e**). IR (CHCl_3_): ν 3606, 3444, 3065, 3019, 2975, 1729, 1675, 1391, 1357, 1215, 1186, 760 cm^–1^. HRMS (FAB+, *m*/*z*): [M]**^+^** calcd for C_22_H_20_F_3_NO_3_, 403.1395; Found, 403.1378.

**3-(Trifluoromethyl)-*N*-{1-(trimethylsilyl)benzyl}glutarimide 3**. To a three-necked round-bottomed flask containing THF (4 mL) and diisopropylamine (0.14 mL, 0.99 mmol), *n*-BuLi (2.6 M: 0.39 mL, 0.99 mmol) was added at −80 °C, and the mixture was stirred for 0.5 h at that temperature. To this solution, 0.0814 g of *N*-benzyl-3-(trifluoromethyl)-glutarimide **1** (0.300 mmol) was added and stirred 0.5 h at the same temperature, and 0.08 mL of TMSCl (0.63 mmol) was introduced, followed by the whole solution being stirred overnight with a gradual increase in the temperature to room temperature. After quenching this reaction with the addition of H_2_O, the crude material was extracted with AcOEt three times, and the combined organic phase was dried over anhydrous Na_2_SO_4_. After filtration, the removal of the volatiles furnished the crude materials, which were purified by silica-gel column chromatography (Hex:AcOEt = 3:1) to afford 0.0378 g (0.110 mmol, 37% yield) of the title compound **3**.

Rf = 0.60 (hexane:AcOEt = 3:1). ^1^H NMR: δ 0.01 (9H, s), 2.63–2.75 (1H, m), 2.68 (1H, dd, *J =* 9.3, 20.1 Hz), 2.77–2.88 (1H, m), 2.91 (1H, dd, *J =* 3.0, 12.6 Hz), 2.97 (1H, td, *J =* 3.0, 16.2 Hz), 4.89 (1H. s), 7.02–7.31 (5H, m). ^13^C NMR: δ –0.83, 31.7, 31.8, 34.0 (q, *J =* 29.8 Hz) 49.9, 125.5 (q, *J =* 277.8 Hz), 126.3, 127.8, 128.3, 139.6, 169.9. ^19^F NMR: δ −75.03 (d, *J* = 6.8 Hz). IR (CHCl_3_): ν 3394, 3029, 2957, 2036, 1373, 1676, 1279, 1249, 1179, 1131, 846, 756, 699 cm^–1^. HRMS (FAB+, *m*/*z*): [M+H]^+^ calcd for C_16_H_21_F_3_NO_2_Si, 344.1294; Found, 344.1309.

***N*-Benzyl-2,4-bis(1-hydroxypropyl)-3-(trifluoromethyl)glutarimide, *anti*,*anti*-4b**. Yield 33% (LHMDS). *dr* = 97:3, Rf = 0.35 (hexane:AcOEt = 3:1). ^1^H NMR: δ 0.95 (6H, t, *J =* 7.4 Hz), 1.49–1.62 (4H, m), 2.86 (2H, d, *J =* 3.3 Hz), 3.12–3.22 (3H, m), 4.09–4.17 (2H, m), 4.99 (2H, s), 7.26–7.30 (5H, m). ^13^C NMR: δ 10.1, 27.6, 33.1 (q, *J =* 27.1 Hz), 44.5, 45.6, 72.5, 127.3 (q, *J =* 278.9 Hz), 127.5, 128.2, 128.4, 136.5, 171.3. ^19^F NMR: δ −72.96 (d, *J =* 9.3 Hz), −71.25 (d, *J =* 9.0 Hz: minor isomer). IR (CHCl_3_): ν 3475, 3020, 2966, 1731, 1673, 1398, 1359, 1217, 1183, 1128, 754 cm^–1^. HRMS (FAB+, m/z): [M]^+^ calcd for C_19_H_24_F_3_NO_4_, 387.1657; Found, 387.1654, [M+H]^+^ calcd for C_19_H_25_F_3_NO_4_, 388.1736; Found, 388.1722.

(2R^*^,3R^*^,4R^*^)-*N*-Benzyl-tetrahydro-2-isopropyl-6-oxo-4-(trifluoromethyl)-2*H*-pyran-3-carboxamide, *syn*,*anti*-5c. Yield 6% (LDA), 34% (LHMDS). dr = >99:<1, Rf = 0.39 (hexane:AcOEt = 1:1), White solid, mp. 182.2 °C. ^1^H NMR: δ 0.97 (3H, d, *J =* 6.6 Hz), 1.11 (3H, d, *J =* 6.6 Hz), 2.02–2.07 (1H, m), 2.75 (1H, dd, *J =* 6.6, 17.7 Hz), 2.86–2.99 (2H, m), 3.13 (1H, dd, *J =* 12.0, 17.4 Hz), 3.93 (1H, d, *J =* 9.3 Hz), 4.37 (1H, dd, *J =* 4.5, 14.1 Hz), 4.45 (1H, dd, *J =* 5.7, 14.4 Hz), 6.39 (1H, s), 7.24–7.34 (5H, m). ^13^C NMR (acetone-*d*_6_): δ 18.9, 19.6, 27.4, 31.5, 38.9, 40.8 (q, *J =* 28.1 Hz), 43.5, 86.3, 126.8 (q, *J =* 226.4 Hz), 127.9, 128.6, 129.0, 139.7, 167.8, 168.6. ^19^F NMR: δ −72.46 (d, *J =* 9.0 Hz). IR (KBr): ν 3317, 3067, 2967, 2933, 2474, 2422, 1725, 1658, 1550, 1270, 1126, 731 cm^–1^. HRMS (FAB+, *m*/*z*): [M]^+^ calcd for C_18_H_22_F_3_NO_3_, 357.1552; Found, 357.1560.

***N*-Benzyl-2-(1-ethyl-1-hydroxypropyl)-3-(trifluoromethyl)glutarimide, 3,7-*syn*-6a**. Yield 36%. **3,7-*syn*-6a**:**3,7-*anti*-6a** = >99: <1, Rf = 0.31 (hexane:AcOEt = 3:1). ^1^H NMR: δ 0.84 (3H, t, *J =* 7.5 Hz), 0.87 (3H, t, *J =* 7.5 Hz), 1.33–1.48 (1H, m), 1.48–1.67 (4H, m), 2.85 (1H, d, *J =* 18.3 Hz) 2.88–3.00 (1H, m), 2.94 (1H, s), 3.29 (1H, dd, *J =* 7.8, 18.6 Hz), 4.95 (1H, d, *J =* 13.8 Hz), 5.04 (1H, d, *J =* 13.8 Hz), 7.05–7.29 (5H, m). ^13^C NMR: δ 7.6, 7.7, 28.7, 29.1, 29.3, 34.9 (q, *J =* 26.4 Hz), 43.3, 46.0, 76.9, 126.9 (q, *J =* 268.6 Hz), 127.3, 128.2, 128.5, 136.8, 170.1, 170.7. ^19^F NMR: δ −73.13 (d, *J =* 11.3 Hz), −72.76 (d, *J* = 11.6 Hz: **3,7-*anti*-6a**). IR (CHCl_3_): ν 3497, 3021, 2974, 2945, 1275, 1676, 1389, 1217, 1147, 747 cm^–1^. HRMS (FAB+, m/z): [M+H]^+^ calcd for C_22_H_22_F_3_NO_3_, 358.1530; Found, 358.1607.

***N*-Benzyl-2-(1-hydroxy-1-methylpropyl)-3-(trifluoromethyl)glutarimide, 3,7-*syn*-6b.** Yield 39%. **3,7-*syn*-6b**:**3,7-*anti*-6b** = 59:41, Rf = 0.30 (hexane:AcOEt = 3:1). ^1^H NMR: δ 0.94 (3H, t, *J =* 6.0 Hz) and 0.92 (3H, t, *J =* 6.0 Hz: **3,7-*anti*-6b**), 1.19 (3H, s: **3,7-*anti*-6b**) and 1.27 (3H, s), 1.43 (1H, qd, 7.2, 13.8 Hz), 1.57–1.70 (2H, m), 2.84–3.02 (3H, m), 3.22–3.34 (1H, m), 4.96 (1H, d, *J =* 13.8 Hz), 5.03 (1H, d, *J =* 14.1 Hz), 7.22–7.33 (5H, m). ^19^F NMR: δ −73.30 (d, *J =* 9.0 Hz) and −73.17 (d, *J* = 9.0 Hz: **3,7-*anti*-6b**).

Data for the diastereomer mixture.

^13^C NMR: δ 8.3, 8.4, 25.5, 26.1, 34.1, 34.4, 34.9 (q, *J* = 27.2 Hz), 35.3 (q, *J* = 27.9 Hz), 43.3, 47.2, 74.8, 126.8 (q, *J =* 277.5 Hz), 127.4, 128.3, 128.5, 128.6, 136.7, 136.8, 169.9, 170.1, 170.6, 170.7. IR (CHCl_3_): ν 3487, 3031, 2976, 2945, 1725, 1671, 1390, 1356, 1226, 1118, 758 cm^–1^. HRMS (FAB+, *m*/*z*): [M]^+^ calcd for C_19_H_20_F_3_NO_3_, 343.1395; Found, 343.1381.

***N*-Benzyl-2-(1-hydroxycyclohex-2-en-1-yl)-3-(trifluoromethyl)glutarimide, 6d**. Yield 11%. *dr =* 68:32, Rf = 0.26 (hexane:AcOEt = 3:1). ^1^H NMR: δ 1.41–1.50 (1H, m), 1.57–1.78 (3H, m), 1.81 (1H, s), 1.99–2.13 (3H, m), 2.89 (1H, d, *J =* 18.9 Hz), 3.02 (1H, s), 3.30 (1H, dd, *J =* 8.1, 18.6 Hz), 5.00 (2H, s), 5.69 (1H, d, *J =* 10.5 Hz), 5.96 (1H, td, *J =* 3.6, 10.2 Hz), 7.21–7.35 (5H, m). ^13^C NMR: δ 19.0, 24.8, 29.2, 34.9 (q, *J =* 14.3 Hz), 35.1, 43.3, 48.0, 71.9, 127.3, 128.2, 128.5, 129.3, 130.1 (q, *J =* 238.8 Hz), 133.2, 136.8, 169.8, 169.9. ^19^F NMR: δ −73.31 (d, *J =* 9.0 Hz), −73.27 (d, *J =* 9.0 Hz: minor isomer). IR (CHCl_3_): ν 3475, 3020, 2930, 1725, 1673, 1391, 1357, 1216, 1193, 1121, 1119, 754 cm^–1^. HRMS (FAB+, m/z): [M]^+^ calcd for C_19_H_20_F_3_NO_3_, 367.1395 ; Found, 367.1406.

**Ethyl 3-{*N*-benzyl-2,6-dioxo-4-(trifluoromethyl)piperidin-3-yl}propanoate, 3,7-*syn*-7a**. Yield 81%. **3,7-*syn*-7a**:**3,7-*anti*-7a** = >99: <1, Rf = 0.29 (hexane:AcOEt = 3:1) ^1^H NMR: δ 1.26 (3H, t, *J =* 7.2 Hz), 2.04–2.12 (2H, m), 2.44 (2H, t, *J =* 7.4 Hz), 2.67–2.70 (1H, m), 2.86–3.05 (3H, m), 4.13 (2H, q, *J =* 7.1 Hz), 4.90 (1H, d, *J =* 13.8 Hz), 4.96 (1H, d, *J =* 13.8 Hz), 7.22–7.32 (5H, m). ^13^C NMR: δ 14.0, 25.7, 28.5, 29.6, 38.7 (q, *J =* 27.9 Hz), 40.1, 43.2, 60.7, 126.0 (q, *J =* 279.9 Hz), 127.5, 128.3, 128.5, 136.5, 168.2, 171.3, 172.1. ^19^F NMR: δ −72.49 (d, *J =* 9.3 Hz), −68.38 (d, *J =* 9.2 Hz: **3,7-*anti*-7a**). IR (CHCl_3_): ν 3027, 2983, 2938, 1730, 1680, 1353, 1221, 1175, 1128, 1033, 559 cm^–1^. HRMS (FAB+, *m*/*z*): [M+H]^+^ calcd for C_18_H_21_F_3_NO_4_, 372.1423; Found, 372.1449.

**Ethyl 3-{*N*-benzyl-2,6-dioxo-4-(trifluoromethyl)piperidin-3-yl}-3-methyl-propanoate, 3,7-*syn*-7b.** Yield 77%. **3,7-*syn*-7b**:**3,7-*anti*-7b** = 69:31, Rf = 0.31 (hexane:AcOEt = 3:1). ^1^H NMR: δ 1.09 (3H, d, *J =* 6.6 Hz), 1.24 (3H, t, *J =* 7.1 Hz), 2.23 (1H, q, *J =* 6.6 Hz), 2.27–2.47 (2H, m), 2.78 (2H, d, *J =* 8.4 Hz), 2.95–2.97 (2H, m), 4.08 (2H, q, *J =* 7.2 Hz), 4.93 (2H, s), 7.21–7.31 (5H, m). ^13^C NMR: δ 13.9, 18.2, 27.8, 30.7, 36.0 (q, *J =* 27.7 Hz), 38.4, 43.1, 45.0, 60.4, 126.6 (q, *J =* 279.7 Hz), 127.2, 128.1, 128.4, 136.4, 168.2, 171.1, 171.5. ^19^F NMR: δ −72.80 (d, *J =* 9.0 Hz), −72.78 (d, *J =* 6.7 Hz: **3,7-*anti*-7b**). IR (CHCl_3_): ν 3033, 2980, 2942, 1732, 1677, 1392, 1354, 1223, 1177, 1127, 1030, 758 cm^–1^. HRMS (FAB+, *m*/*z*): [M+H]^+^ calcd for C_22_H_22_F_3_NO_3_, 386.1579; Found, 386.1598.

**Ethyl 3-{*N*-benzyl-2,6-dioxo-4-(trifluoromethyl)piperidin-3-yl}-3-phenyl-propanoate, 3,7-*syn*-7c.** Yield 63%. **3,7-*syn*-7c**:**3,7-*anti*-7c** = 53:47, Rf = 0.41 (hexane:AcOEt = 3:1).

Data for the diastereomer mixture.

^1^H NMR: δ 1.18 (3H, t, *J =* 7.1 Hz), 1.74 (1H, dd, *J =* 7.5, 18.3 Hz), 2.55 (1H, d, *J =* 18.6 Hz), 2.66 (1H, m), 2.77 (1H, dd, *J =* 6.6, 16.8 Hz), 3.16–3.21 (2H, m), 3.57–3.78 (2H, m), 4.85 (1H, d, *J =* 13.5 Hz), 4.96 (1H, d, *J =* 13.8 Hz), 5.04 (1H, s), 7.18–7.78 (10H, m). ^13^C NMR: δ 13.9, 27.8, 31.2, 34.0 (q, *J =* 29.3 Hz), 38.8, 39.1 (q, *J =* 27.7 Hz), 42.6, 43.0, 46.3, 60.6, 77.2, 126.1 (q, *J* = 267.1 Hz), 127.4, 127.6, 127.7, 128.0, 128.3, 128.3, 128.9, 129.0, 129.3, 130.0, 130.3, 134.7, 136.1, 136.5, 138.4, 141.4, 168.1, 168.5, 170.1, 171.5, 171.6. ^19^F NMR: δ −73.64 (d, *J =* 9.0 Hz), −73.59 (d, *J* = 9.0 Hz). IR (CHCl_3_): ν 3064, 3029, 2982, 1725, 1676, 1632, 1435, 1410, 1174, 1119, 760 cm^–1^. HRMS (FAB+, *m*/*z*): [M+H]^+^ calcd for C_24_H_25_F_3_NO_4_, 448.1736; Found, 448.1727.

***N*-Benzyl-2-(3-phenylpropan-3-on-1-yl)-3-(trifluoromethyl)glutarimide, 3,7-*syn*-7d.** Yield 52%. **3,7-*syn*-7d**:**3,7-*anti*-7d** = 87:13, Rf = 0.27 (hexane:AcOEt = 3:1). ^1^H NMR: δ 2.11–2.28 (2H, m), 2.68–2.78 (1H, m), 2.91–3.13 (3H, m), 3.08 (2H, t, *J =* 7.5 Hz), 4.95 (2H, s), 7.20–7.91 (10H, m). ^13^C NMR: δ 25.2, 28.3, 35.2, 38.8 (q, *J* =2 7.9 Hz), 40.1, 43.1, 126.1 (q, *J =* 278.0 Hz), 127.5, 127.9, 128.3, 128.5, 128.6, 133.4, 136.2, 136.5, 168.4, 171.7, 198.2. ^19^F NMR: δ −72.71 (d, *J =* 9.0 Hz), −71.19 (d, *J =* 9.0 Hz: **3,7-*anti*-7d**). IR (CHCl_3_): ν 3065, 3029, 2960, 1731, 1681, 1393, 1353, 1223, 1175, 1127, 756 cm^–1^. HRMS (FAB+, *m*/*z*): [M+2H]^+^ calcd for C_22_H_22_F_3_NO_3_, 405.1552; Found, 405.1581.

**3-{*N*-Benzyl-2,6-dioxo-4-(trifluoromethyl)piperidin-3-yl}-*N*-phenylbutanamide, 3,7-*syn*-7e**. Yield 88%. **3,7-*syn*-7e:3,7-*anti*-7e =** >99:<1, Rf = 0.11 (hexane:AcOEt =3:1). ^1^H NMR (acetone-*d*_6_): δ 1.88 (3H, d, *J=*6.3 Hz), 2.39 (1H, dd, *J =* 7.5, 16.2 Hz), 2.56 (1H, dd, *J =* 5.7, 15.6 Hz), 2.65–2.70 (1H, m), 2.81–2.93 (2H, m), 3.24–3.38 (2H, m), 4.83 (1H, d, *J =* 14.7 Hz), 4.88 (1H, d, *J =* 14.7 Hz), 7.10–7.65 (10H, m), 9.17 (1H, s). ^13^C NMR: δ 18.6, 27.6, 30.8, 36.5 (q, *J =* 27.7 Hz), 41.1, 43.3, 45.4, 119.7, 124.3, 126.0 (q, *J =* 280.0 Hz), 127.4, 128.3, 128.3, 128.9, 136.4, 137.6, 168.4, 168.8, 172.0. ^19^F NMR: δ −72.66 (d, *J =* 9.0 Hz). IR (CHCl_3_): ν 3223, 3065, 3018, 2971, 1729, 1676, 1601, 1543, 1219, 1177, 1128, 760 cm^–1^. HRMS (FAB+, *m*/*z*): [M]^+^ calcd for C_23_H_23_F_3_N_2_O_3_, 432.1661; Found, 432.1680.

***N*-Benzyl-2-(cyclohexan-1-on-3-yl)-3-(trifluoromethyl)glutarimide, 3,7-*anti*-7g.** Yield 35%. **3,7-*syn*-7f**:**3,7-*anti*-7f** *= >*99: <1, White solid, mp. 134.9 °C, Rf = 0.14 (hexane:AcOEt = 3:1). ^1^H NMR: δ 1.42 (1H, d, *J =* 8.4 Hz), 1.48 (1H, d, *J =* 9.3 Hz), 1.52–1.63 (1H, m), 2.00–2.02 (1H, m), 2.28 (2H, t, *J =* 12.3 Hz), 2.36 (1H, s), 2.43 (2H, dd, *J =* 4.8, 10.2 Hz), 2.71 (1H, t, *J =* 7.8 Hz), 2.80 (1H, d, *J =* 8.4 Hz), 2.90 (1H, dd, *J =* 1.2, 6.3 Hz), 2.94 (1H, s), 4.91 (1H, d, *J =* 13.8 Hz), 5.07 (1H, d, *J =* 13.5 Hz), 7.24–7.32 (5H, m). ^13^C NMR (acetone-*d*_6_): δ 24.8, 28.6, 33.0, 36.2 (q, *J =* 27.1 Hz), 38.9, 41.2, 43.5, 45.6, 46.8, 127.9, 128.0, 128.1 (q, *J* = 279.3 Hz), 129.0, 138.1, 169.5, 171.7, 208.4. ^19^F NMR: δ –72.71 (d, *J =* 9.0 Hz), **–**68.53 (d, *J =* 9.0 Hz: **3,7-*anti*-7f**). IR (KBr): ν 3033, 2955, 2925, 1962, 1705, 1474, 1651, 1497, 1225, 1203, 736 cm^–1^. HRMS (FAB+, *m*/*z*): [M+H]^+^ calcd for C_19_H_21_F_3_NO_3_, 368.1474; Found, 368.1481.

## 4. Conclusions

In this article, we successfully achieved the preferential formation of 3,7-*anti* aldol products by reacting the CF_3_-containing imide **1** with appropriate aldehydes, simply by adding HMPA to the standard conditions, which is nicely compared with the results of our previous studies, affording the corresponding products with 3,7-*syn* selectivity. It is apparent that the realization of these consequences should be due to these reactions proceeding via the non-chelation mechanism by the action of this additive. Moreover, we also employed ketones as well as α,β-unsaturated carbonyl compounds as electrophiles because their applications were not attempted previously, and better outcomes were obtained by THF alone as a solvent for the construction of new carbon–carbon bonds with good to excellent yields and stereoselectivities. Thus, the scope of aldol reactions of **1** was nicely expanded, and it is our desire that these products introduce new synthetic applications to synthesize a variety of efficient compounds, which would lead to the development of useful materials for our life.

## Figures and Tables

**Table 1:** Optimization of the reaction conditions.

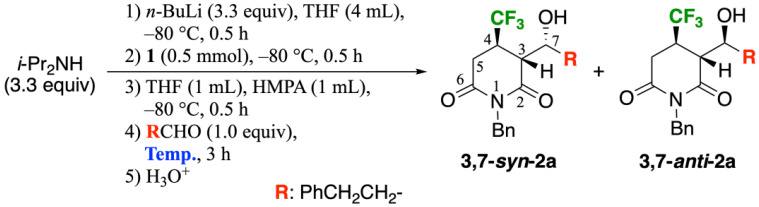						
Entry	Temp. (°C)	^19^F NMR Yield (%)	*syn*:*anti*			Recovery ^(a)^ (%)
1 ^(b)^	–80	71	76	:	24	27
2	–80	(57) ^(c)^	16	:	84	27
3	–60	55	55	:	45	10
4	–40	complex mixture				8
5 ^(d)^	–80	61	56	:	44	29
6 ^(d)^	–80	54	50	:	50	24
7 ^(e)^	–80	45	64	:	36	29
8 ^(e)^	–80	71	75	:	25	24
9 ^(f)^	–80	(75) ^(c)^	16	:	84	6

(a) Determined by ^19^F NMR. (b) 5 mL of THF was added in step 1 with canceling step 3. (c) Isolated yield. (d) THF:HMPA = 10:1 (Entry 5) and 7.5:1 (Entry 6) in their volume. (e) 1,3-dimethyl-2-imidazolidinone (DMI, Entry 7) and tetramethylethylenediamine (TMEDA, Entry 8) were used instead of HMPA. (f) The reaction was carried out for 5 h by using a 2.0 equivalent of RCHO and LHMDS instead of LDA as a base.

**Table 2:** Scope and limitation of the present reactions by using LDA and LHMDS as a base.

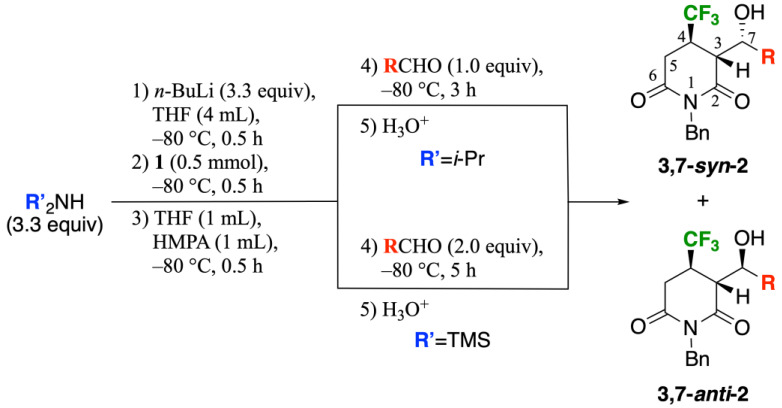					
Entry	R	LDA		LHMDS	
		Yield (%) ^(a)^	dr ^(b)^	Yield (%) ^(a)^	dr ^(b)^
1	Ph(CH_2_)_2_ (a)	57 [27]	16:84	75 [6]	11:89
2	Et (b)	60 [34]	6:94	52 ^(c)^ [4]	8:92
3	*i-*Pr (c)	28 ^(d)^ [37]	39:61	17 ^(d)^ [10]	<1:>99
4	*t-*Bu (d)	60 [37]	7:93	26 [35]	23:77
5	Ph (e)	25 [64]	18:82	32 [35]	12:88
6	*p-*MeOC_6_H_4_ (f)	18 [80]	<1:>99	31 [52]	2:98
7	*p-*BrC_6_H_4_ (g)	30 [42]	19:81	46 [39]	12:88

(a) Isolated yields are shown and the recovery of **1** is shown in the brackets. (b) Diastereomer ratios, *syn*:*anti*, determined by ^19^F NMR. (c) The byproduct **4b** was obtained in 33% yield. (d) The byproduct ***syn*,*anti*-5c** was obtained in 6% (LDA) and 34% (LHMDS) yields.

**Table 3:** Reaction of **1** with ketones and α,β-unsaturated carbonyl compounds.

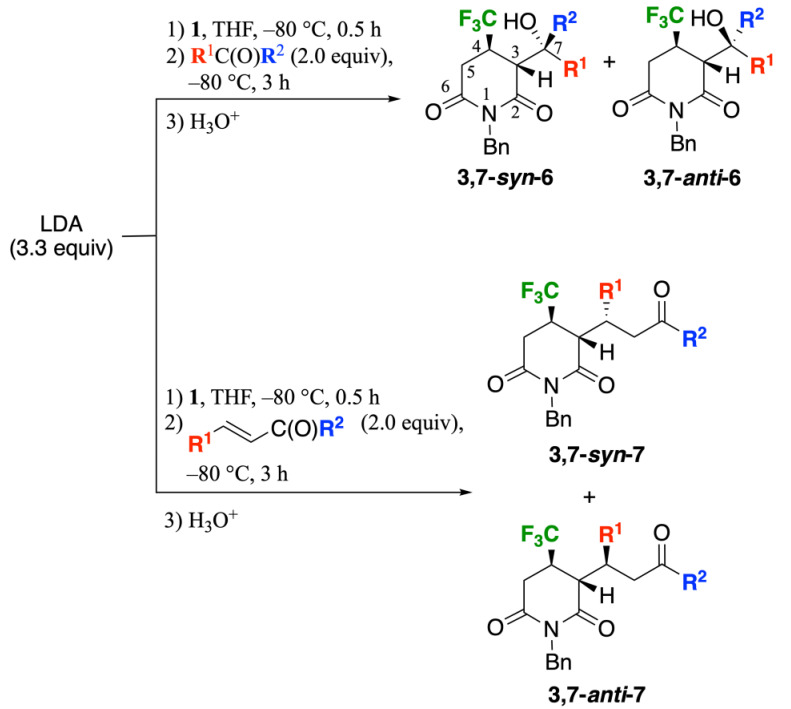				
Entry	R^1^	R^2^	Isolated Yield ^(a)^ (%)	*syn*:*anti* ^(b)^
1	Et	Et	36 [63] **(6a)**	>99:<1
2	Me	Et	39 [60] **(6b)**	59:41
3	Me	Ph	<1 [92] **(6c)**	–
4	H	OEt	81 **(7a)**	>99:<1
5	Me	OEt	77 **(7b)**	69:31
6	Ph	OEt	63 **(7c)**	53:47
7 ^(c)^	H	Ph	52 **(7d)**	87:13
8	H	NHBn	0 [96] **(7e)**	–
9	Ph	H	0 ^(d)^ [81] **(7f)**	–

(a) The numbers in brackets described the recovery of **1** determined by ^19^F NMR. (b) Determined by ^19^F NMR. (c) Reaction was continued for 5 h in step 2. (d) The aldol product **3,7-*syn*-2h** was obtained in 15% yield in an *anti*:*syn* = 62:38 ratio.

## Data Availability

Data are contained within the article and Supplementary Materials.

## Supplementary Materials

The following supporting information can be downloaded at: https://www.mdpi.com/article/10.3390/molecules29215129/s1. ^1^H and ^13^C NMR Spectra for the new compounds.
