# Supplementary material for: Stereodivergent Synthesis of Aldol Products Using Pseudo-C2 Symmetric N-benzyl-4-(trifluoromethyl)piperidine-2,6-dione
Source: Molecules. 2024 Oct 30;29(21):5129. doi: 10.3390/molecules29215129 (PMC11547649; doi:10.3390/molecules29215129)

Supporting Information

Stereodivergent synthesis of aldol products using pseudo C_2 symmetric *N*-benzyl-4-(trifluoromethyl)piperidine-2,6-dione

Rina Yada, Tomoko Kawasaki-Takasuka, and Takashi Yamazaki*

Division of Applied Chemistry, Institute of Engineering, Tokyo University of Agriculture and Technology, 2-24-16 Nakamachi, Koganei 184-8588, Japan

*Correspondence: tyamazak@cc.tuat.ac.jp

^{1}H and ^{13}C NMR Charts for new compounds

(CDCl$_3$ was used as a solvent unless otherwise noted)

(2*R**,3*R**)-*N*-Benzyl-2-{(*S**)-1-hydroxy-3-phenylpropyl}-3-(trifluoromethyl)-glutarimide (**3,7-*anti*-2a**) ········ 3

(2*R**,3*R**)-*N*-Benzyl-2-{(*S**)-1-hydroxypropyl}-3-(trifluoromethyl)glutarimide (**3,7-*anti*-2b**) ········ 4

(2*R**,3*R**)-*N*-Benzyl-2-{(*S**)-1-hydroxy-2-methylpropyl}-3-(trifluoromethyl)-glutarimide (**3,7-*anti*-2c**) ········ 5

(2*R**,3*R**)-*N*-Benzyl-2-{(*S**)-1-hydroxy-2,2-dimethylpropyl}-3-(trifluoromethyl)-glutarimide (**3,7-*anti*-2d**) ········ 6

(2*R**,3*R**)-*N*-Benzyl-2-{(*S**)-1-hydroxy-1-phenylmethyl}-3-(trifluoromethyl)-glutarimide (**3,7-*anti*-2e**) ········ 7

(2*R**,3*R**)-*N*-Benzyl-2-{(*S**)-1-hydroxy-(4-methoxyphenyl)methyl)}-3-(trifluoro-methyl)glutarimide (**3,7-*anti*-2f**) ········ 8

(2*R**,3*R**)-*N*-Benzyl-2-{(*S**)-1-(4-bromophenyl)-1-hydroxymethyl}-3-(trifluoro-methyl)glutarimide (**3,7-*anti*-2g**) ········ 9

N-Benzyl-2-{(*E*)-1-hydroxy-3-phenylprop-2-en-1-yl}-3-(trifluoromethyl)glutarimide
(**3,7-*syn*-2h**) ········ 10
3-(Trifluoromethyl)-*N*-{1-(trimethylsilyl)benzyl}glutarimide (**3**) ········ 11
N-Benzyl-2,4-bis(1-hydroxypropyl)-3-(trifluoromethyl)glutarimide
(***anti*,*anti*-4b**) ········ 12
(2*R**,3*R**,4*R**)-*N*-Benzyl-tetrahydro-2-isopropyl-6-oxo-4-(trifluoromethyl)-2*H*-pyran-3-carboxamide (3,4-***syn*-4,5-*anti*-5c**) ········ 13
N-Benzyl-2-(1-ethyl-1-hydroxypropyl)-3-(trifluoromethyl)glutarimide (**3,4-*anti*-6a**) ········ 14
N-Benzyl-2-(1-hydroxycyclohex-2-en-1-yl)-3-(trifluoromethyl)glutarimide
(**3,7-*syn*-6b**) ········ 15
N-Benzyl-2-(1-hydroxycyclohex-2-en-1-yl)-3-(trifluoromethyl)glutarimide
(**3,4-*anti*-6d**) ········ 16
Ethyl 3-{*N*-benzyl-2,6-dioxo-4-(trifluoromethyl)piperidin-3-yl}propanoate
(**3,4-*anti*-7a**) ········ 17
Ethyl 3-{*N*-benzyl-2,6-dioxo-4-(trifluoromethyl)piperidin-3-yl}-3-methylpropanoate
(**3,7-*syn*-7b**) ········ 18
Ethyl 3-{*N*-benzyl-2,6-dioxo-4-(trifluoromethyl)piperidin-3-yl}-3-phenylpropanoate
(**3,7-*syn*-7c**) ········ 19
N-Benzyl-2-(3-phenylpropan-3-on-1-yl)-3-(trifluoromethyl)glutarimide
(**3,4-*anti*-7d**) ········ 20
N-Benzyl-2-(cyclohexan-1-on-3-yl)-3-(trifluoromethyl)glutarimide
(**3,4-*anti*-7g**) ········ 21

(2*R,3*R**)-*N*-Benzyl-2-{(*S**)-1-hydroxy-3-phenylpropyl}-3-(trifluoromethyl)-glutarimide (3,7-*anti*-2a)**

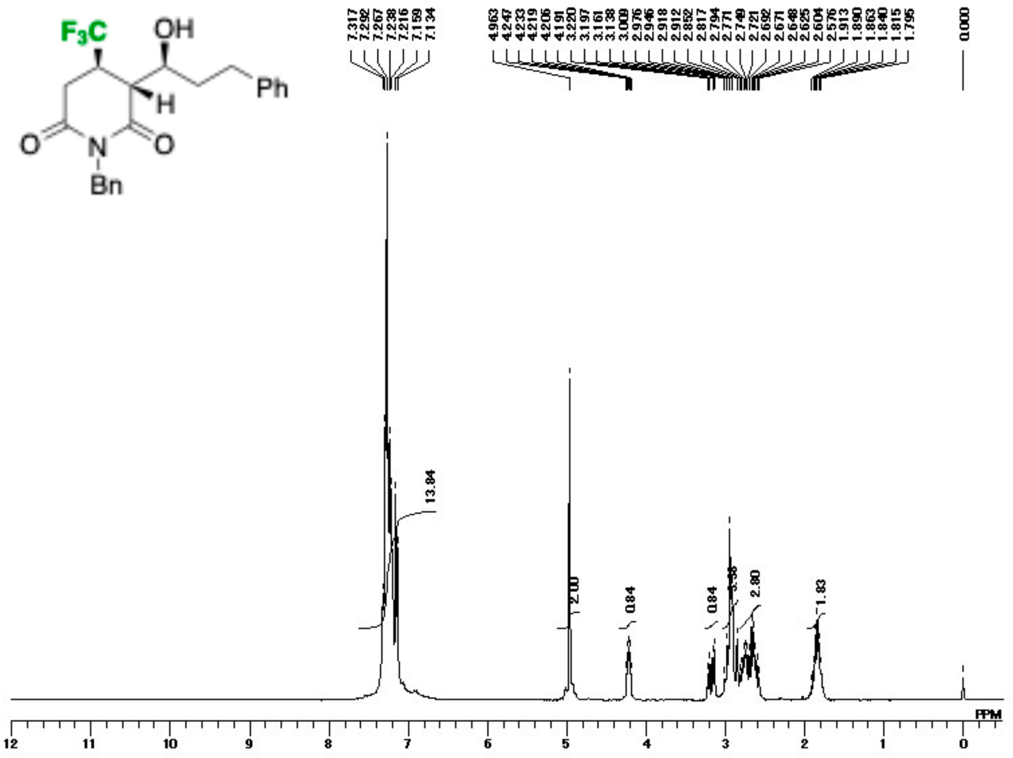

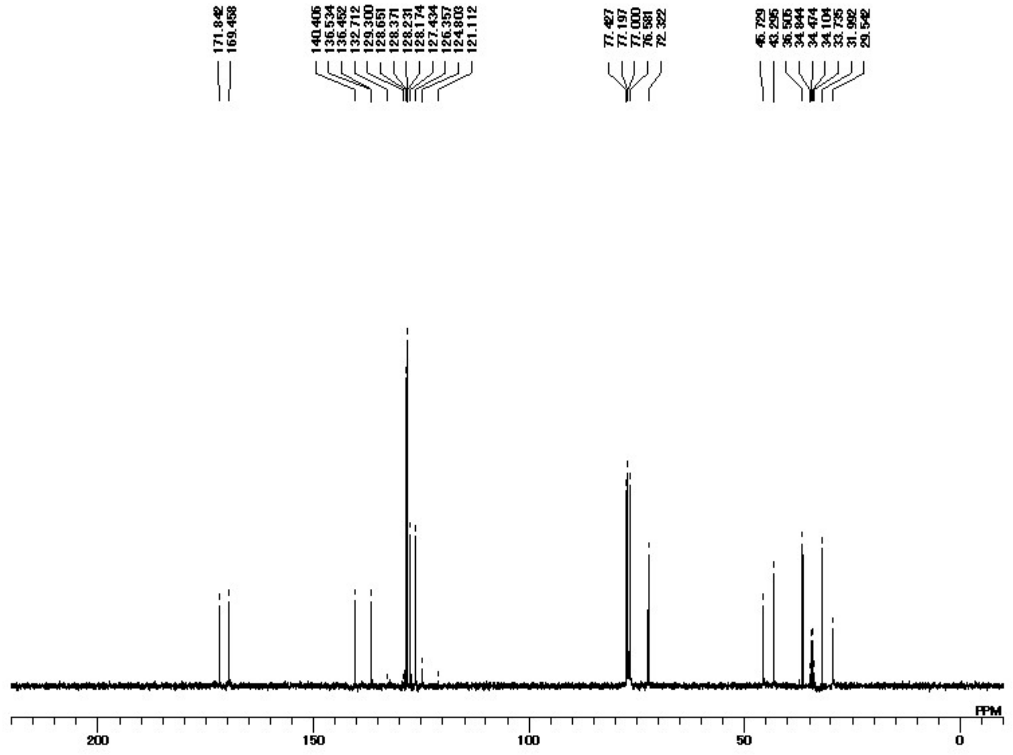

(2*R*[*],3*R*[*])-*N*-Benzyl-2-{(*S*[*])-1-hydroxypropyl}-3-(trifluoromethyl)glutarimide (3,7-*anti*-2b)

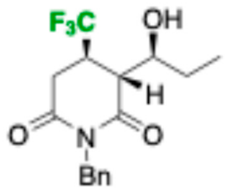

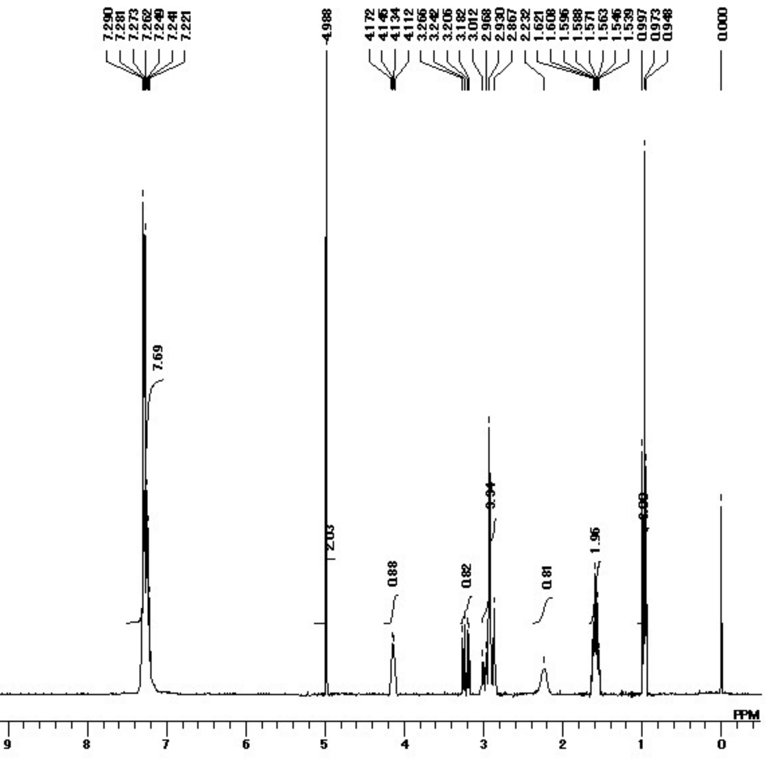

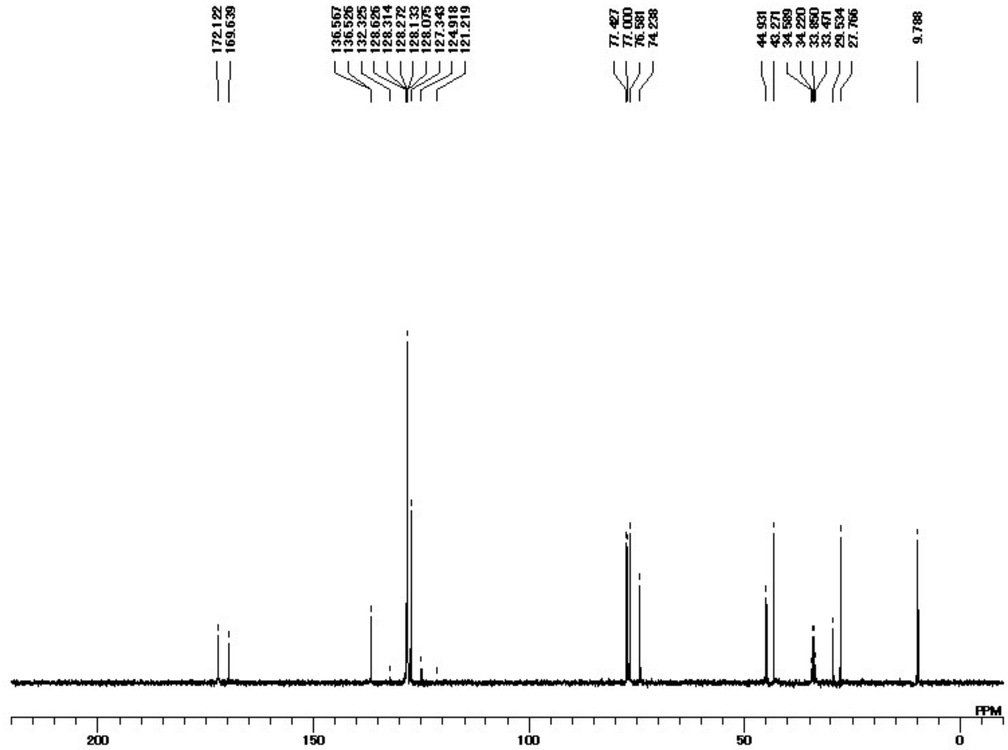

(2*R*[*],3*R*[*])-*N*-Benzyl-2-{(*S*[*])-1-hydroxy-2-methylpropyl}-3-(trifluoromethyl)-glutarimide (3,7-*anti*-2c)

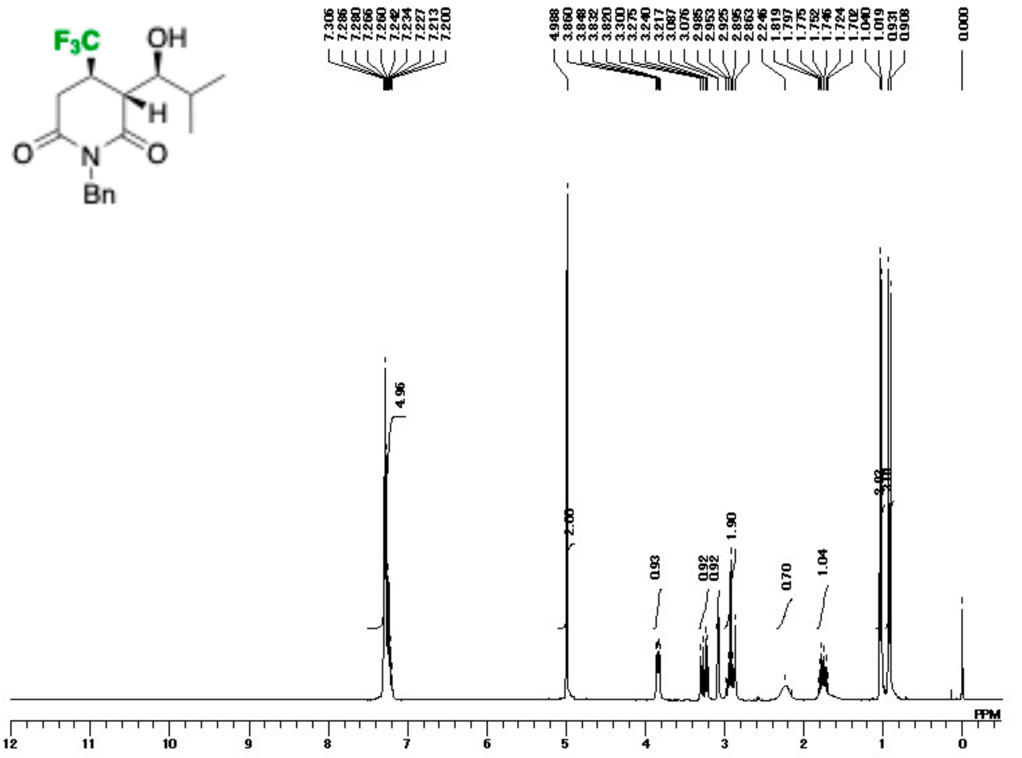

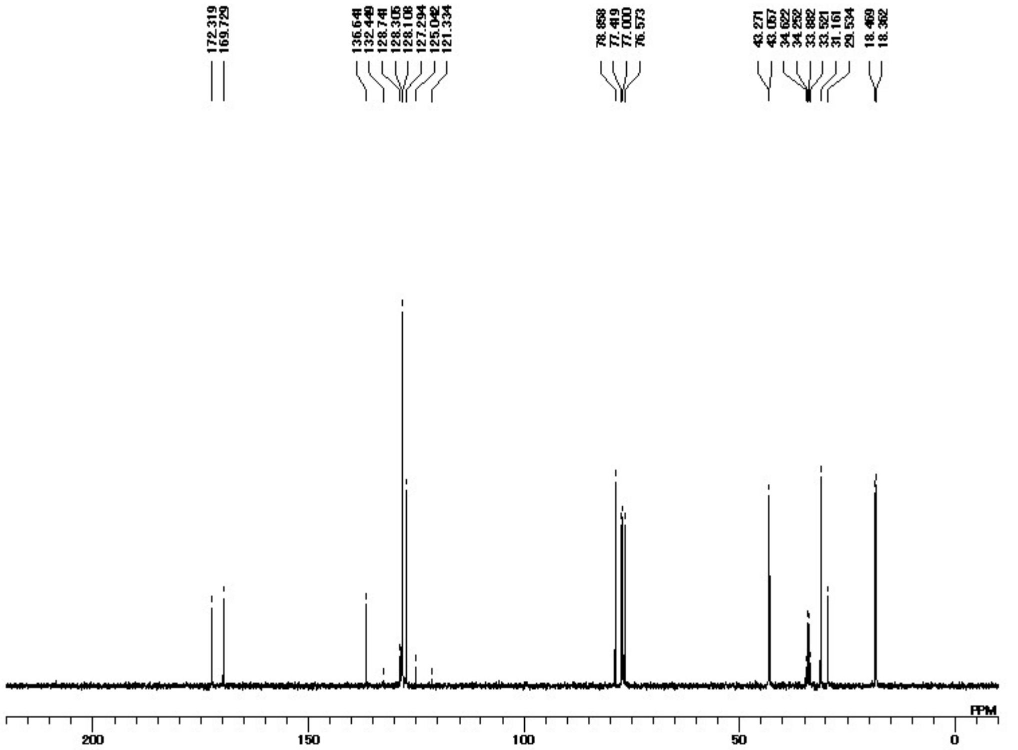

(2*R,3*R**)-*N*-Benzyl-2-{(*S**)-1-hydroxy-2,2-dimethylpropyl}-3-(trifluoromethyl)-glutarimide (3,7-*anti*-2d)**

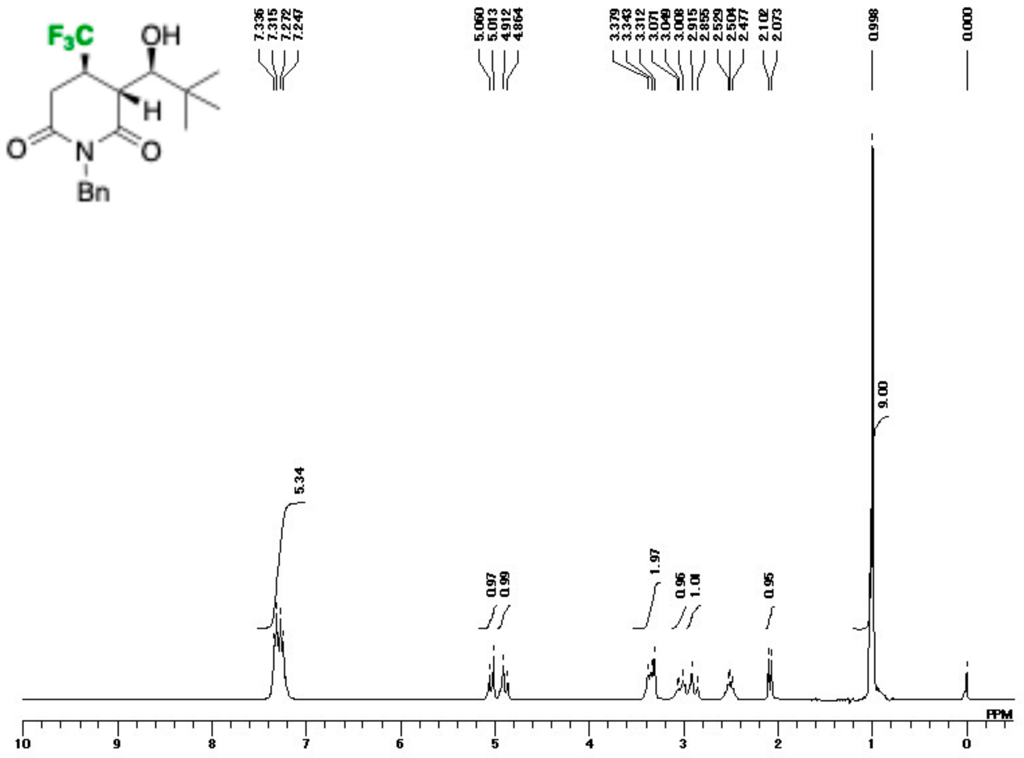

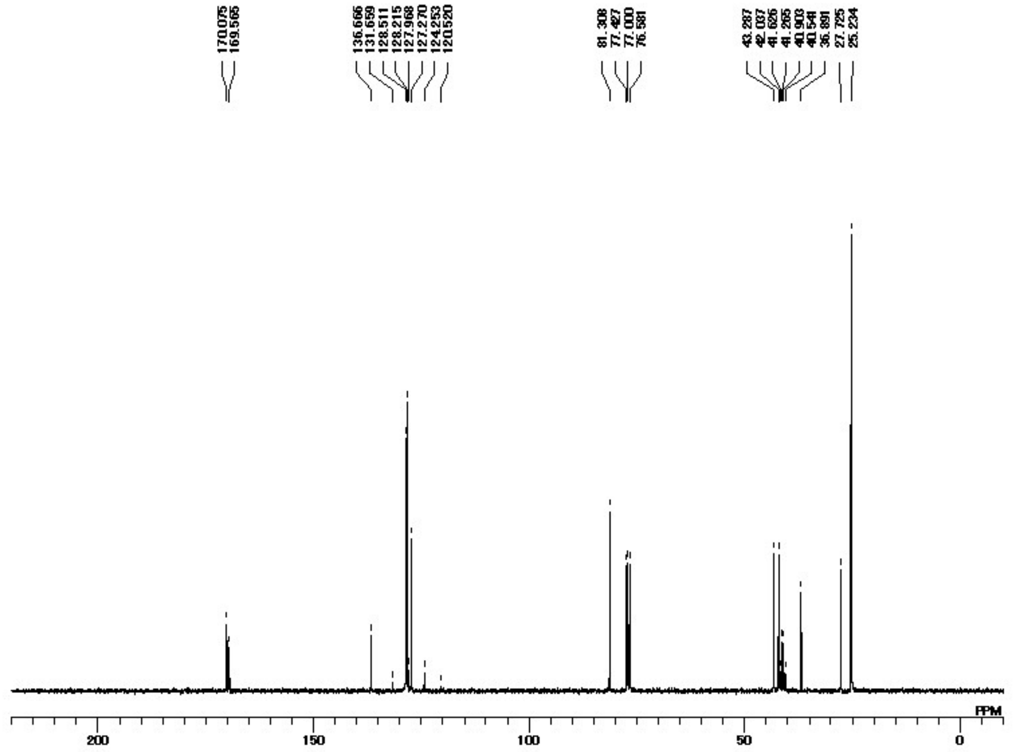

(2R^*,3R^*)-*N*-Benzyl-2-{(S^*)-1-hydroxy-1-phenylmethyl}-3-(trifluoromethyl)-glutarimide (3,7-*anti*-2e)

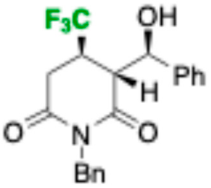

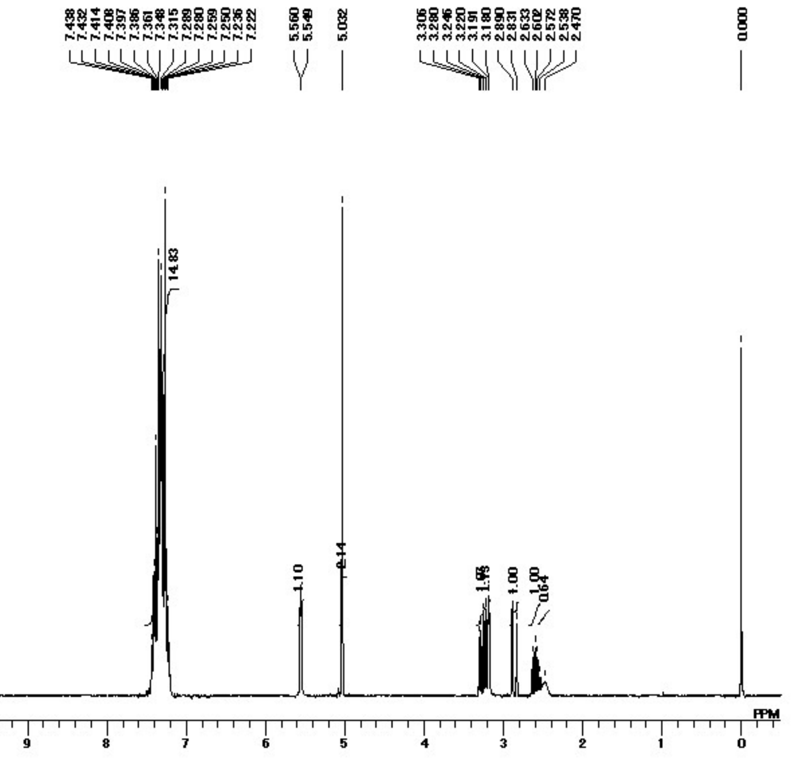

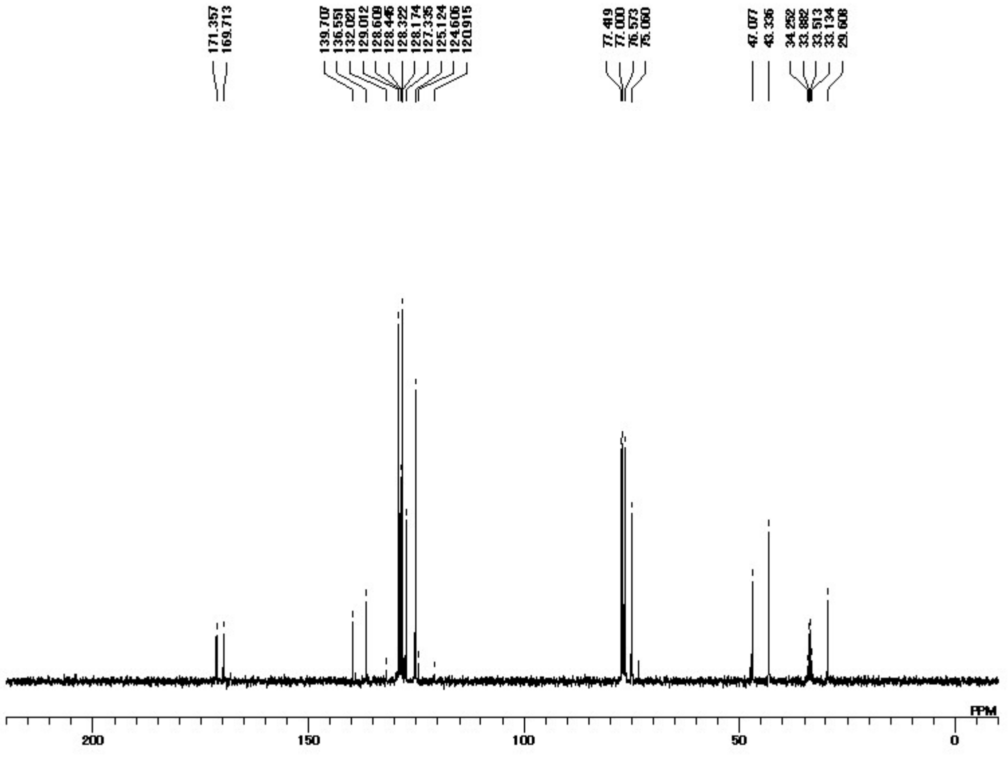

(2*R*[*],3*R*[*])-*N*-Benzyl-2-{(*S*[*])-1-hydroxy-(4-methoxyphenyl)methyl)}-3-(trifluoro-methyl)glutarimide (3,7-*anti*-2f)

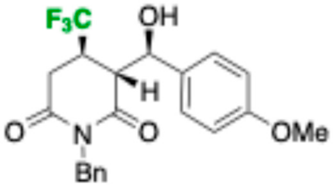

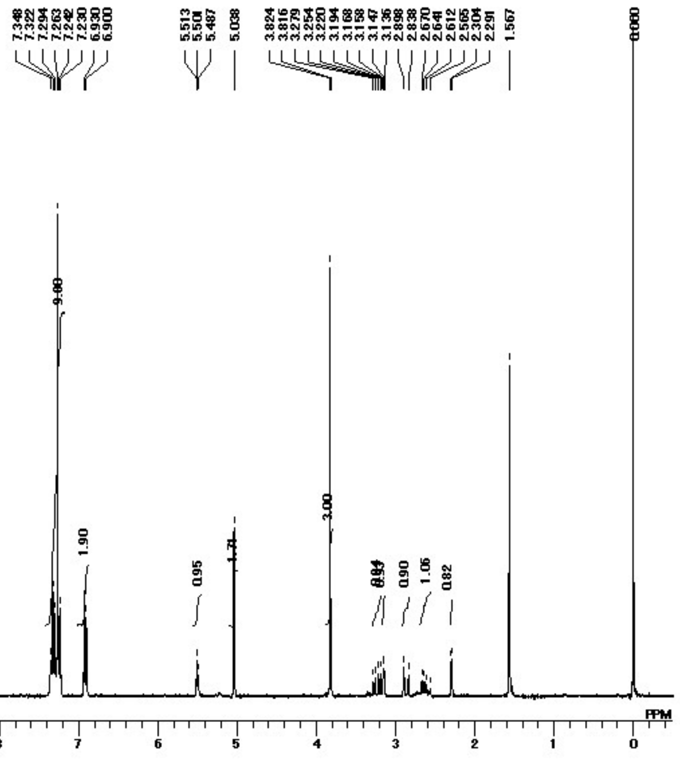

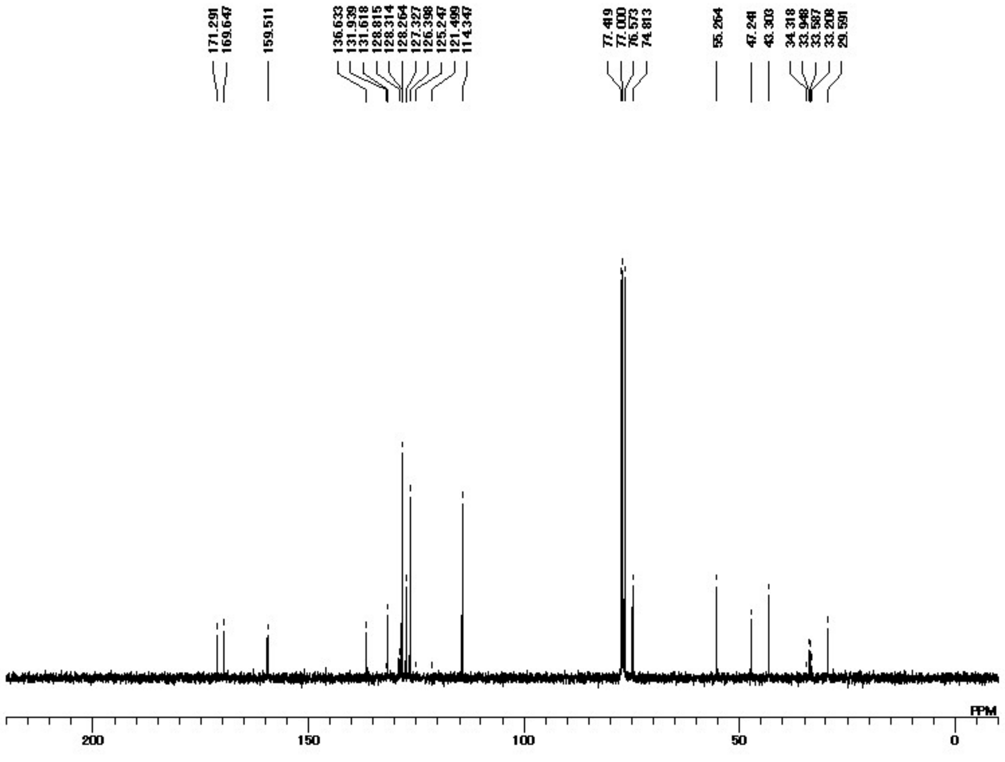

(2R^*,3R^*)-*N*-Benzyl-2-{(S^*)-1-(4-bromophenyl)-1-hydroxymethyl}-3-(trifluoromethyl)glutarimide (3,7-*anti*-2g)

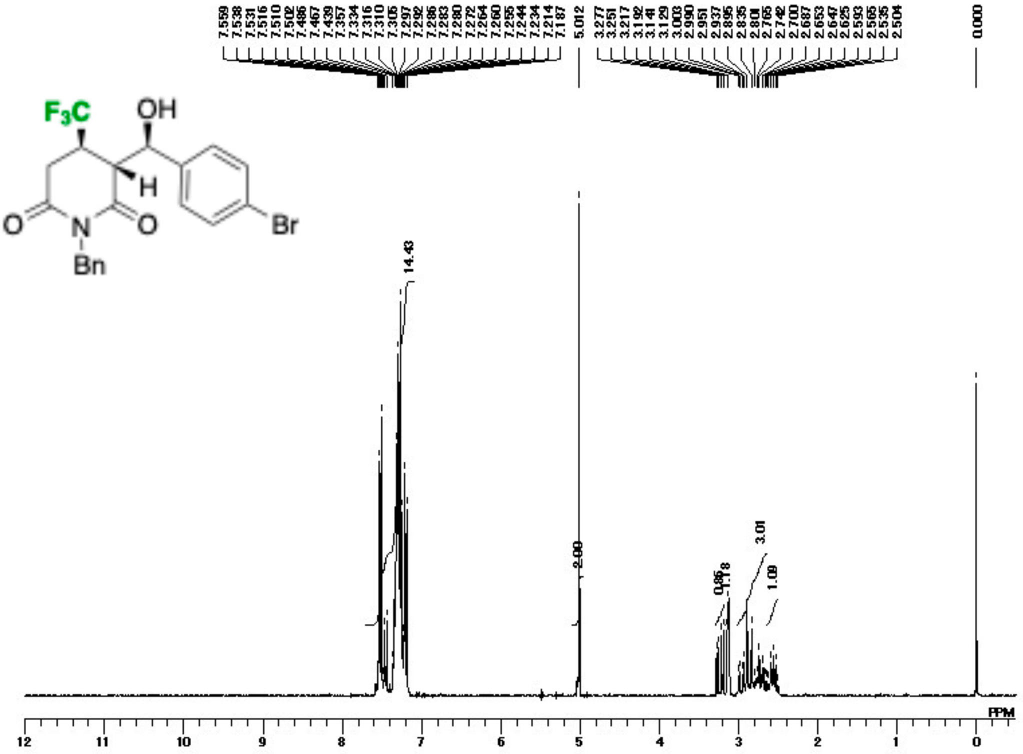

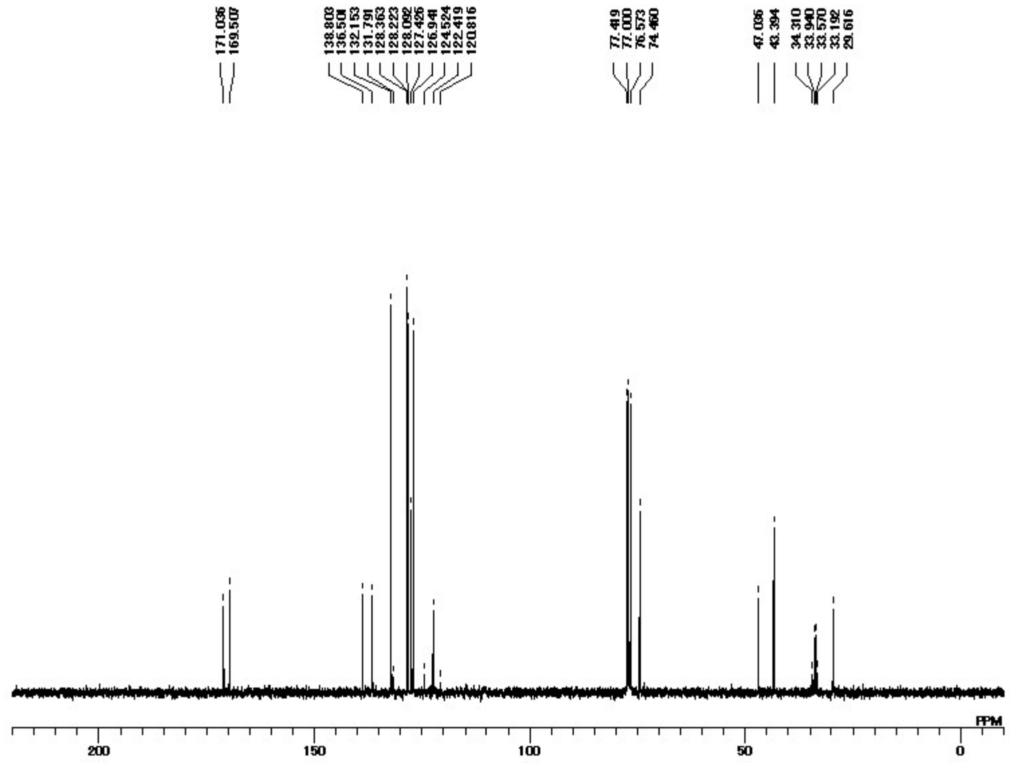

***N*-Benzyl-2-{(*E*)-1-hydroxy-3-phenylprop-2-en-1-yl}-3-(trifluoromethyl)-glutarimide (3,7-*syn*-2h)**

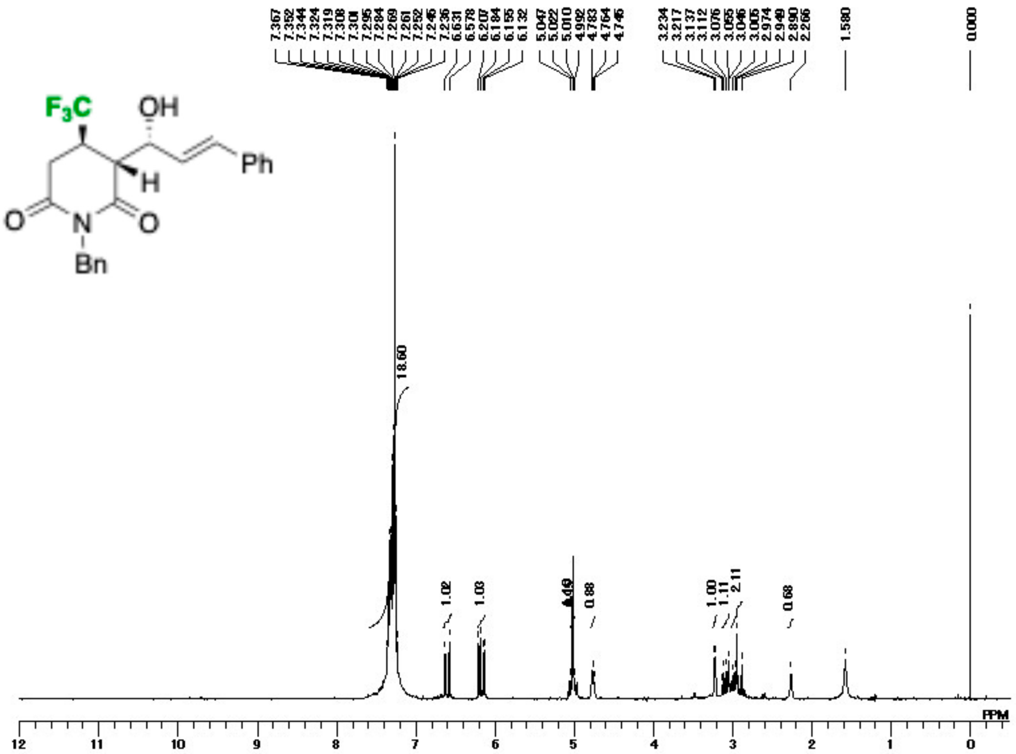

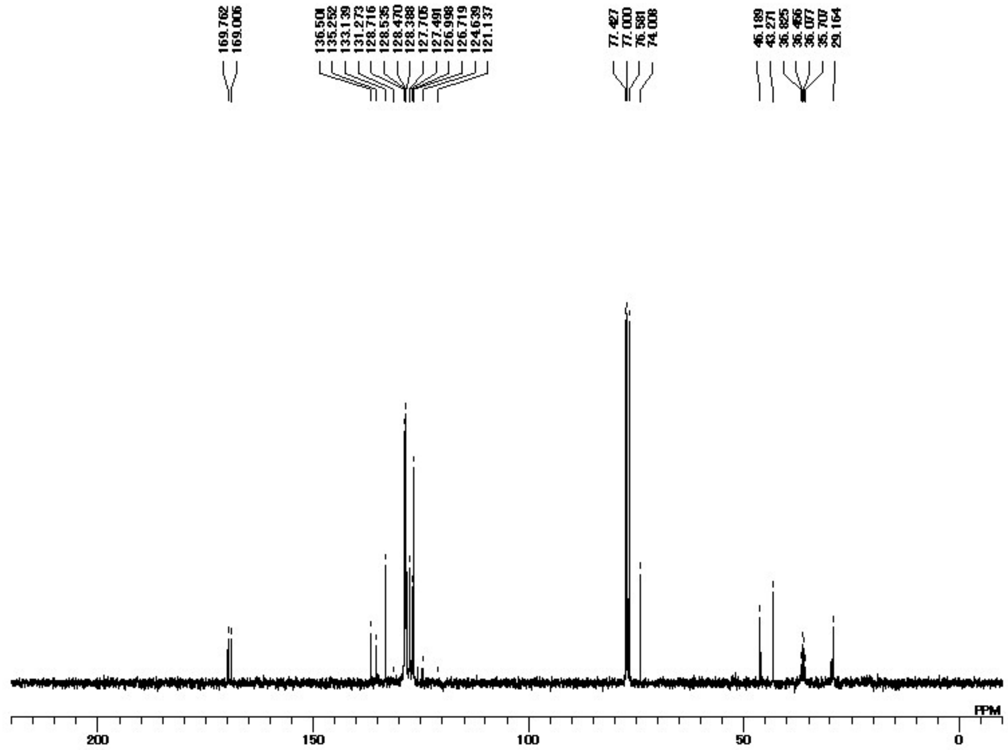

3-(Trifluoromethyl)-*N*-{1-(trimethylsilyl)benzyl}glutarimide (3: ^{1}H NMR was observed in acetone-d_6 as a solventy)

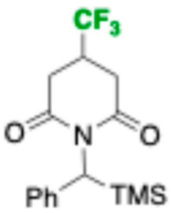

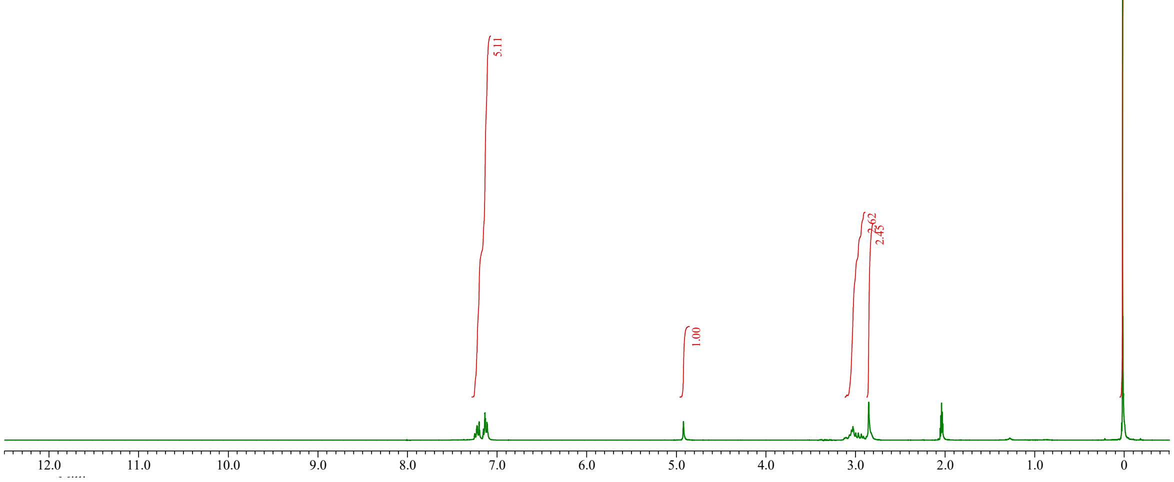

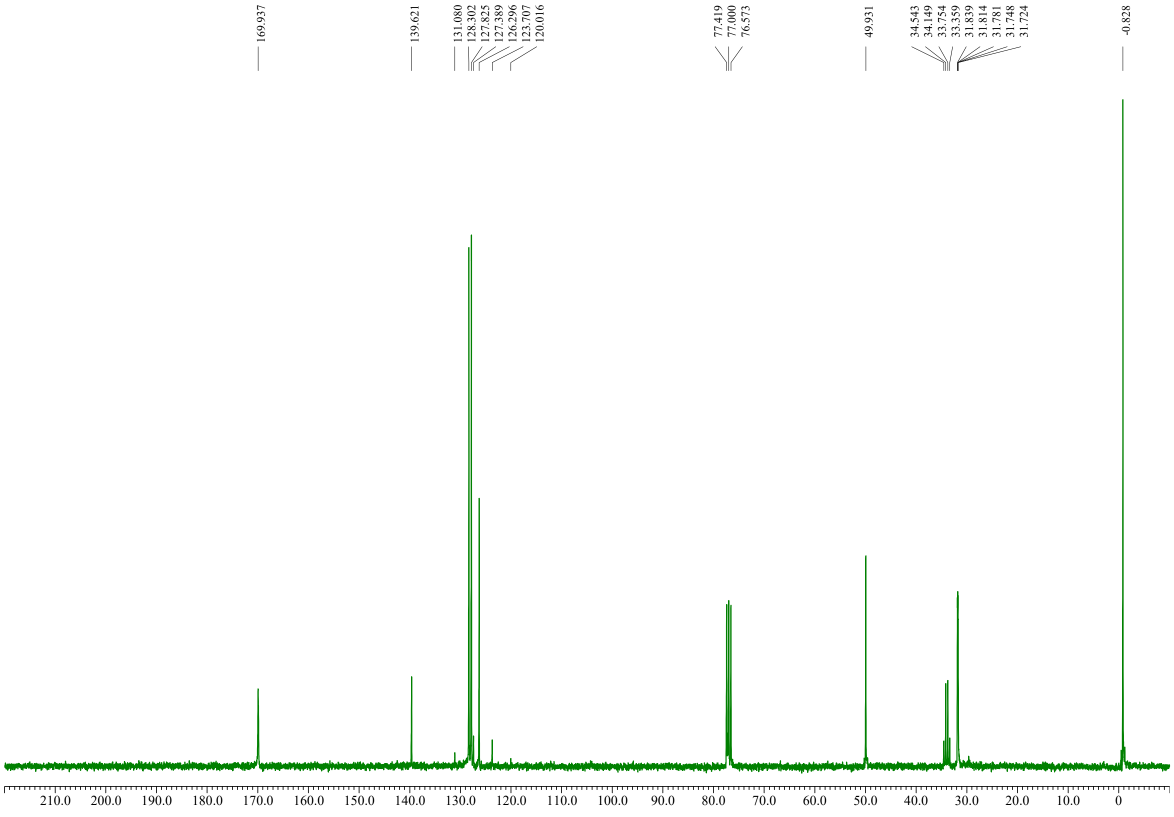

_N_-Benzyl-2,4-bis(1-hydroxypropyl)-3-(trifluoromethyl)glutarimide (_anti_,_anti_-4b)

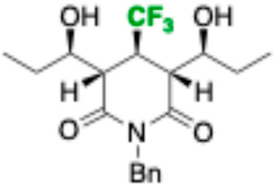

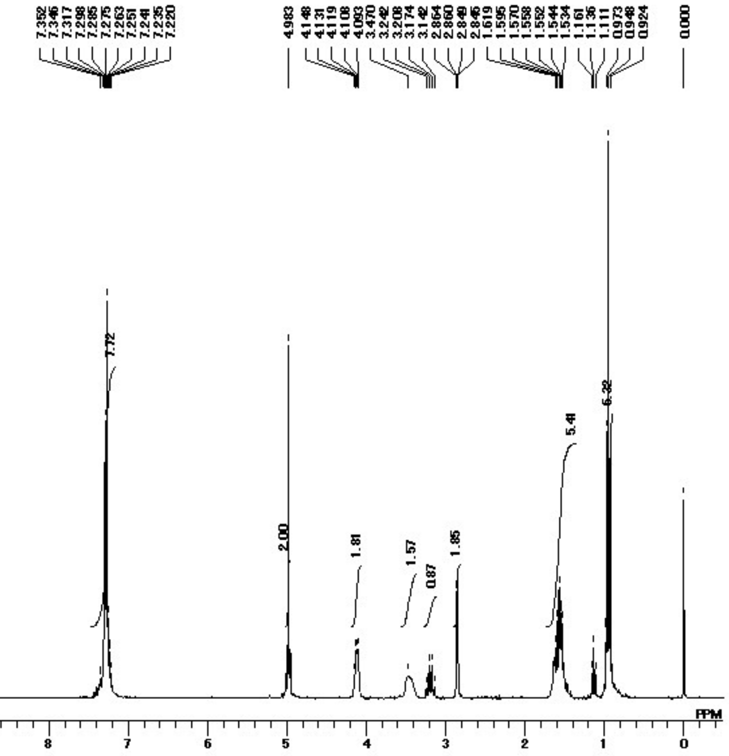

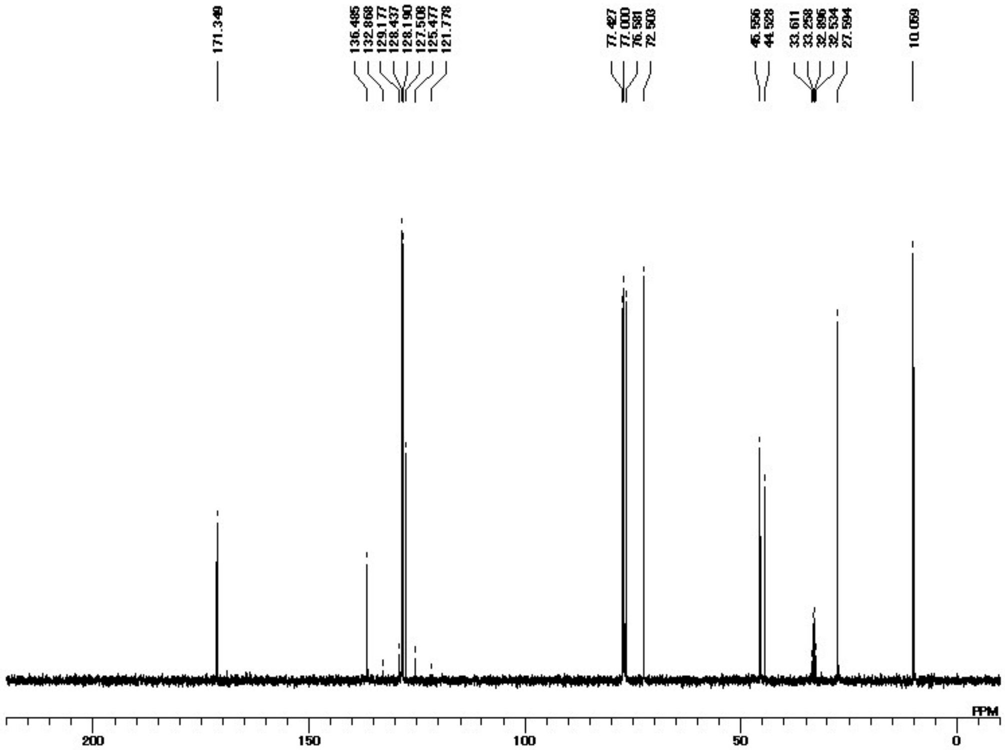

(2*R*,3*R***,4*R***)-*N*-Benzyl-tetrahydro-2-isopropyl-6-oxo-4-(trifluoromethyl)-2*H*-pyran-3-carboxamide (*syn*,*anti*-5c)**

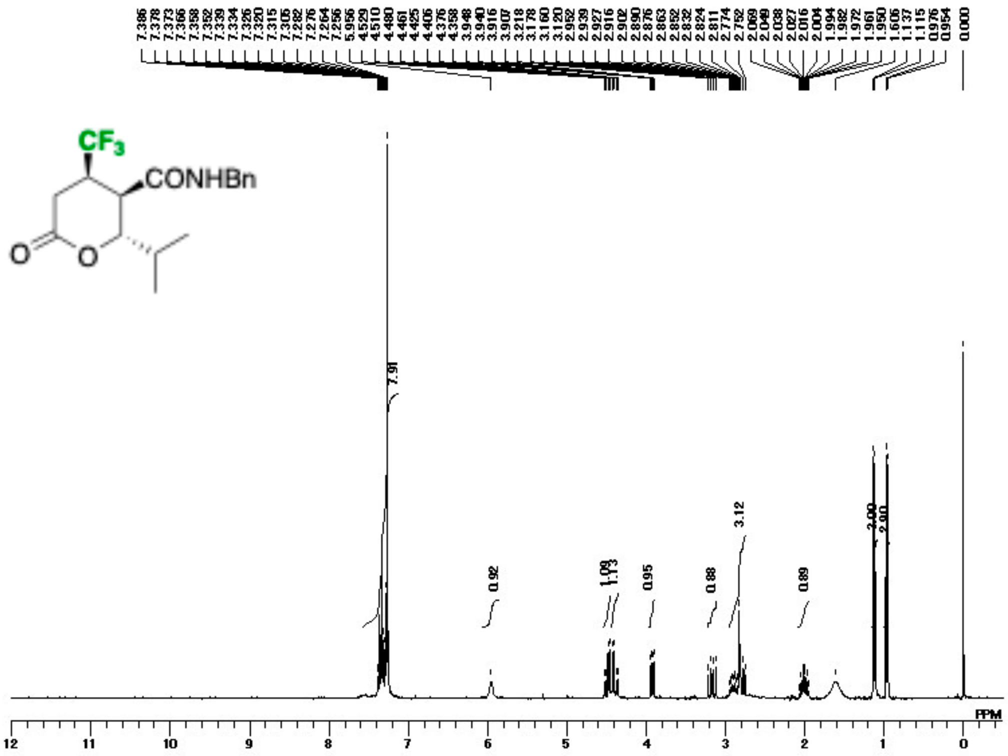

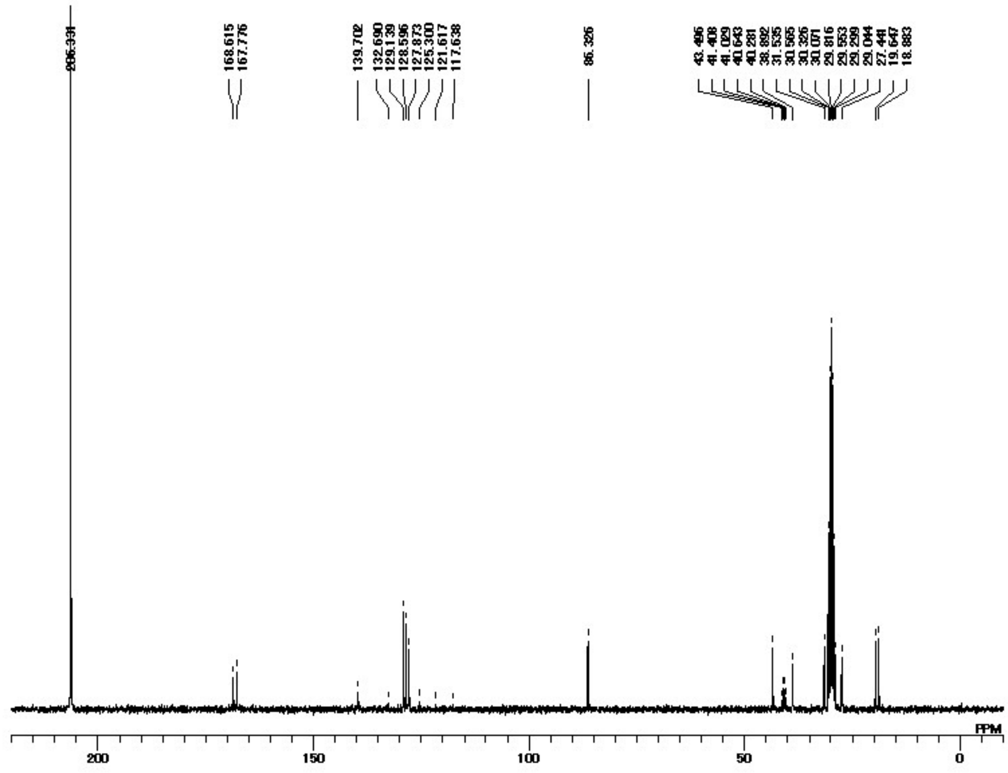

***N*-Benzyl-2-(1-ethyl-1-hydroxypropyl)-3-(trifluoromethyl)glutarimide (3,4-*anti*-6a)**

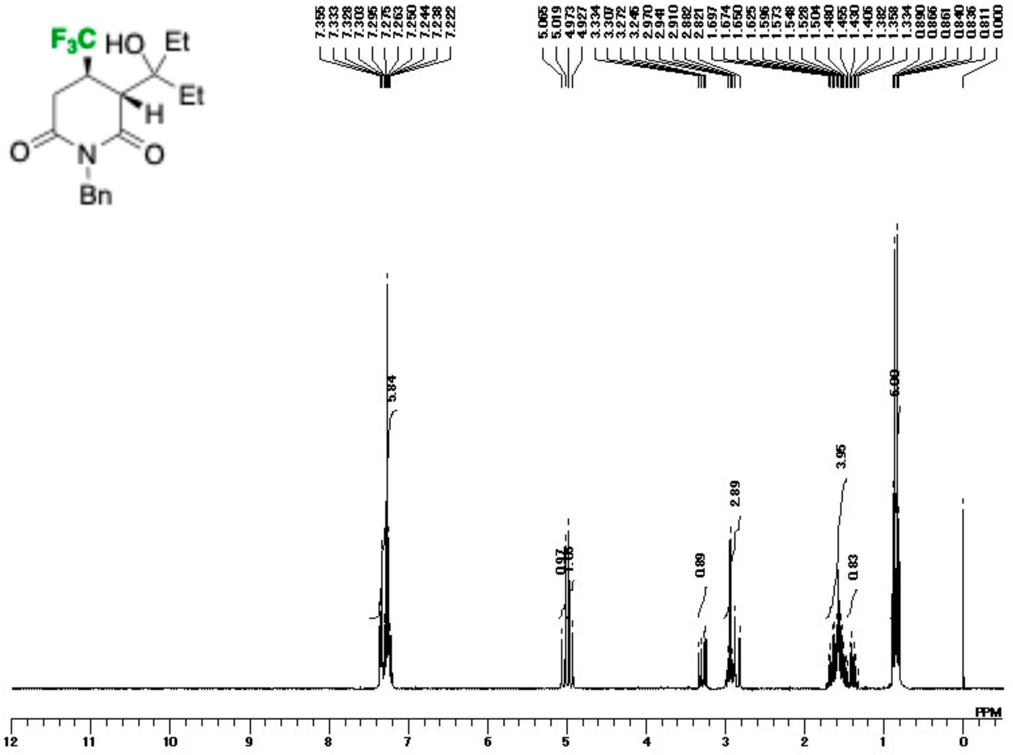

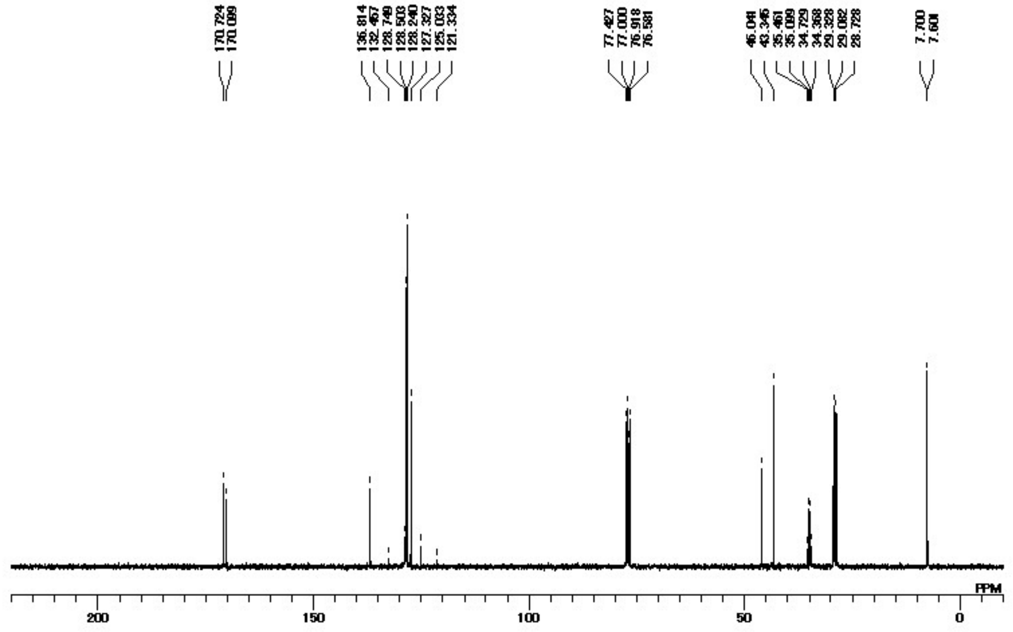

***N*-Benzyl-2-(1-hydroxycyclohex-2-en-1-yl)-3-(trifluoromethyl)glutarimide (3,7-*syn*-6b)**

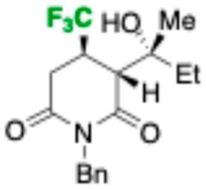

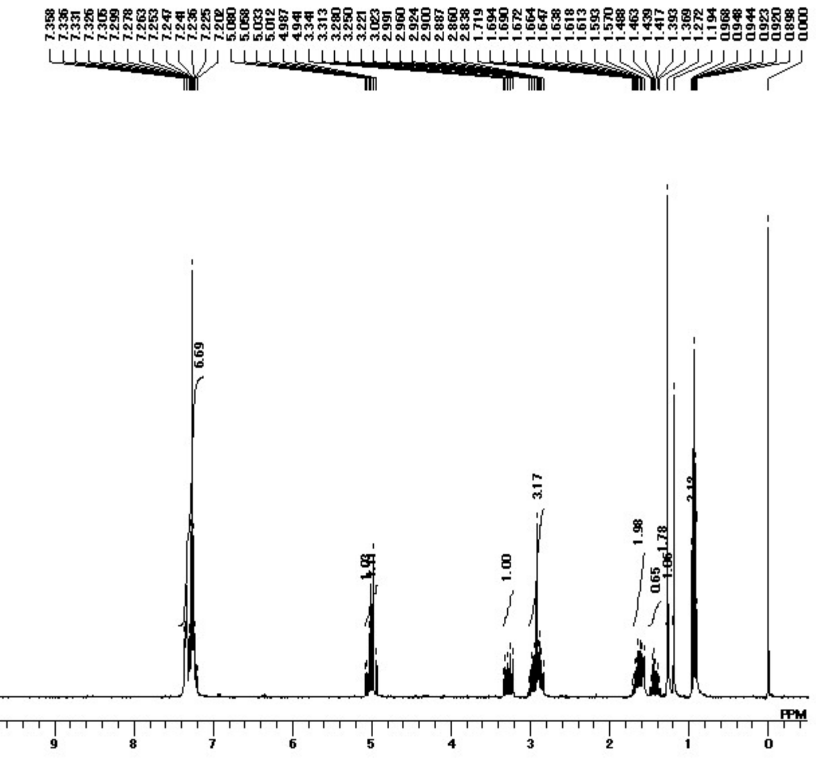

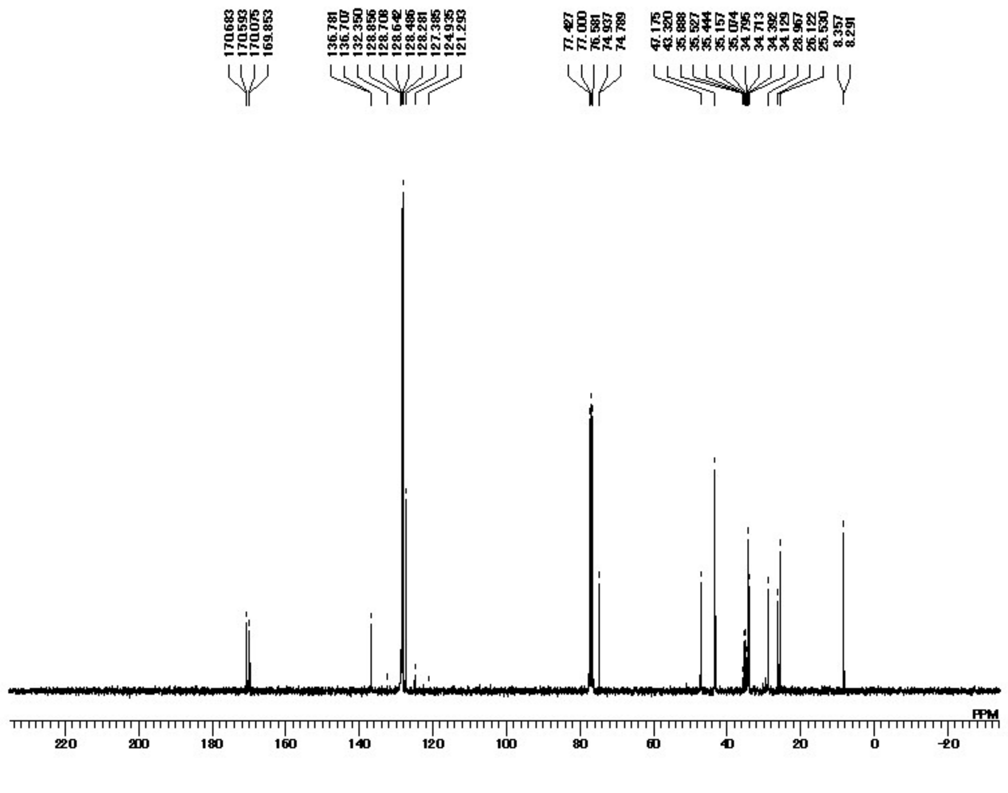

***N*-Benzyl-2-(1-hydroxycyclohex-2-en-1-yl)-3-(trifluoromethyl)glutarimide (3,4-*anti*-6d)**

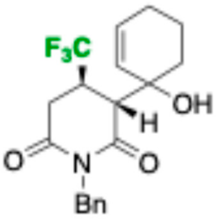

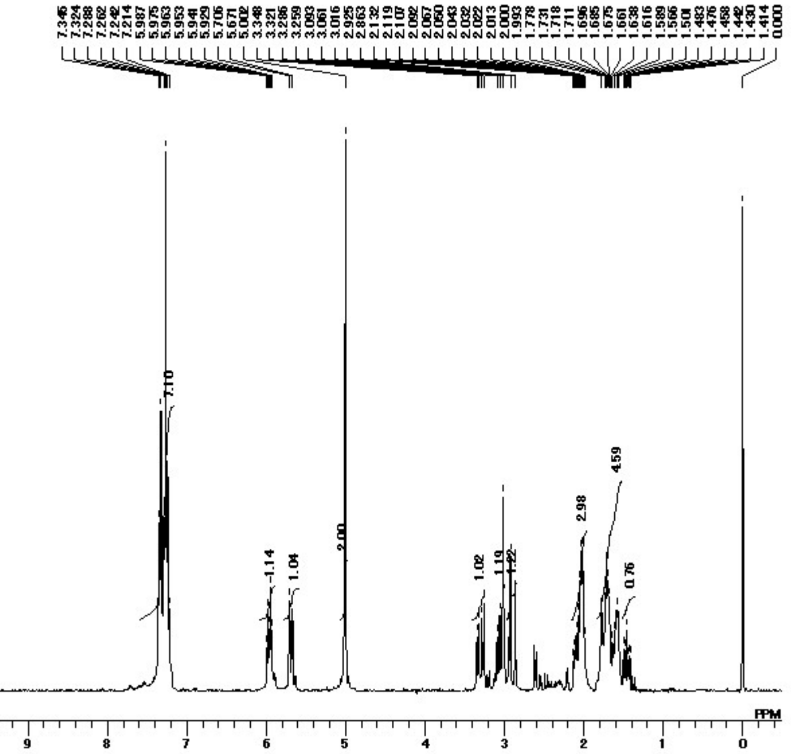

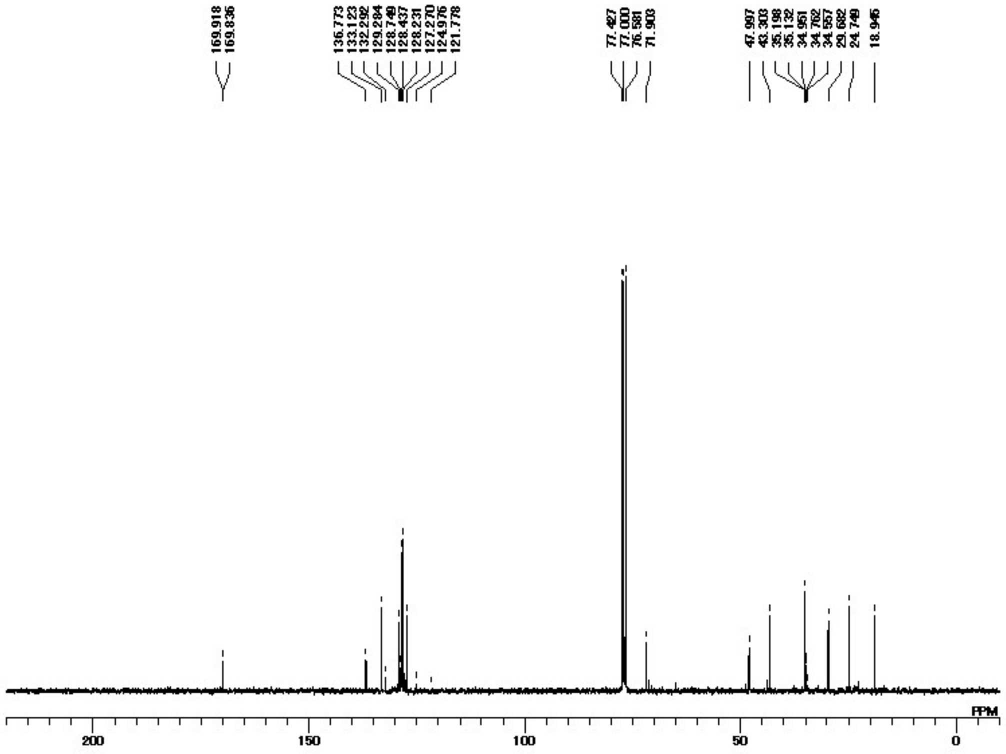

Ethyl 3-{*N*-benzyl-2,6-dioxo-4-(trifluoromethyl)piperidin-3-yl}propanoate (3,4-*anti*-7a)

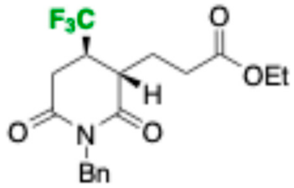

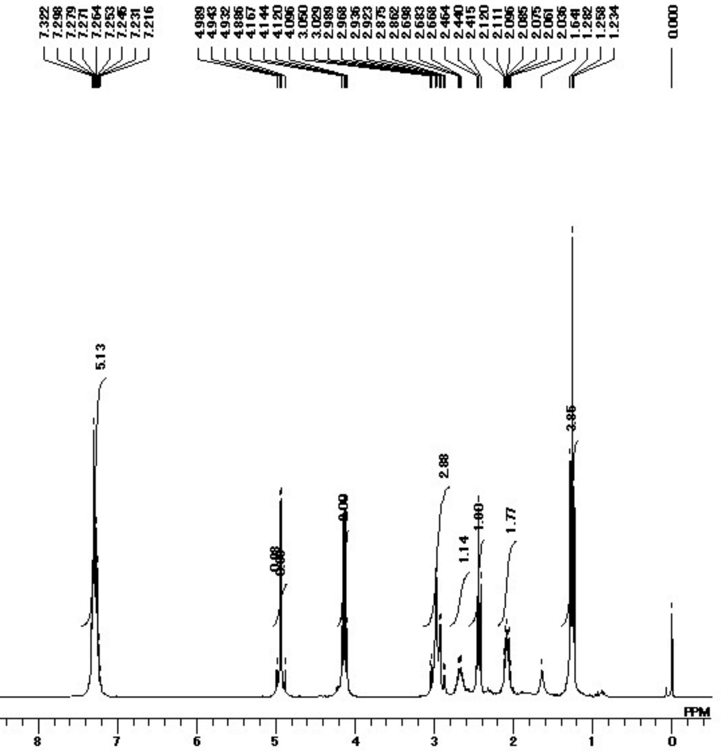

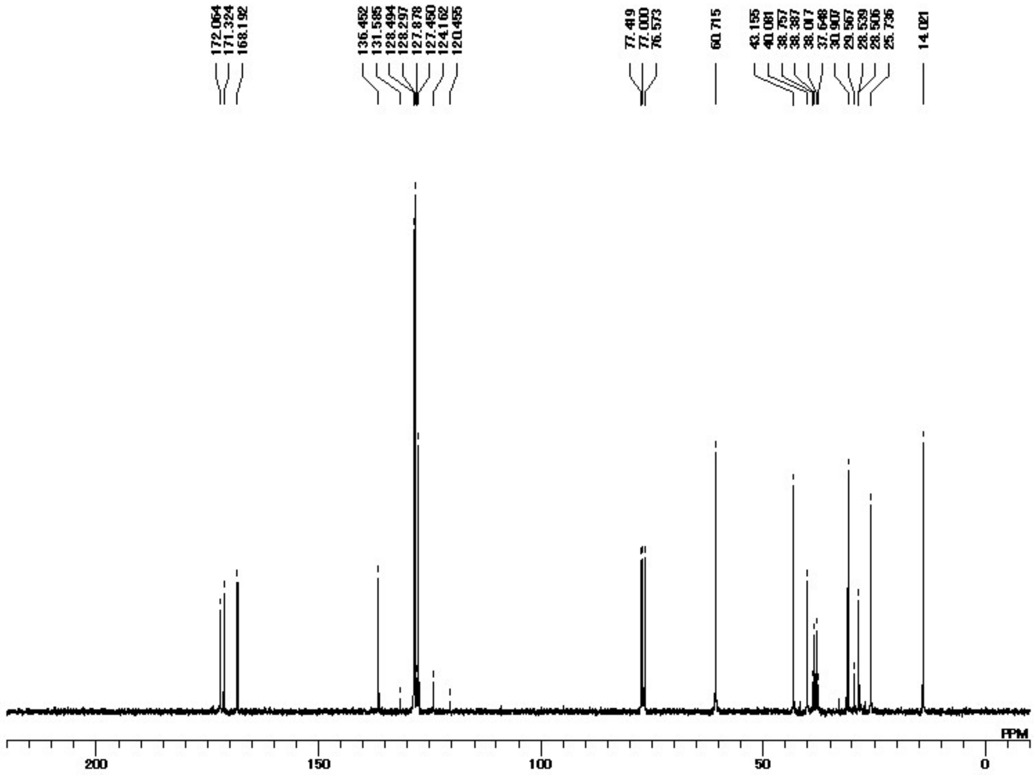

Ethyl 3-{*N*-benzyl-2,6-dioxo-4-(trifluoromethyl)piperidin-3-yl}-3-methyl-propanoate (3,7-*syn*-7b)

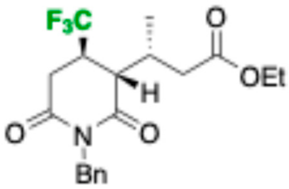

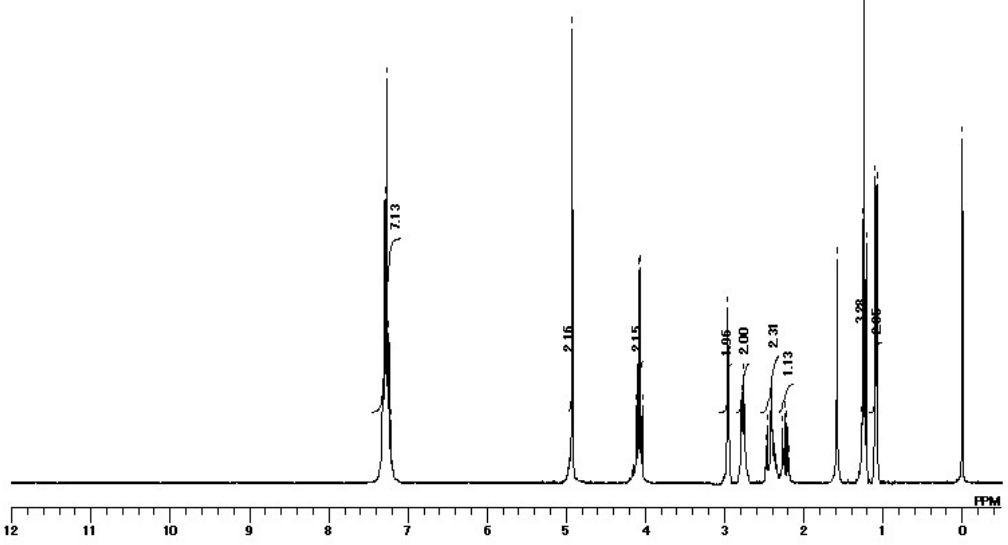

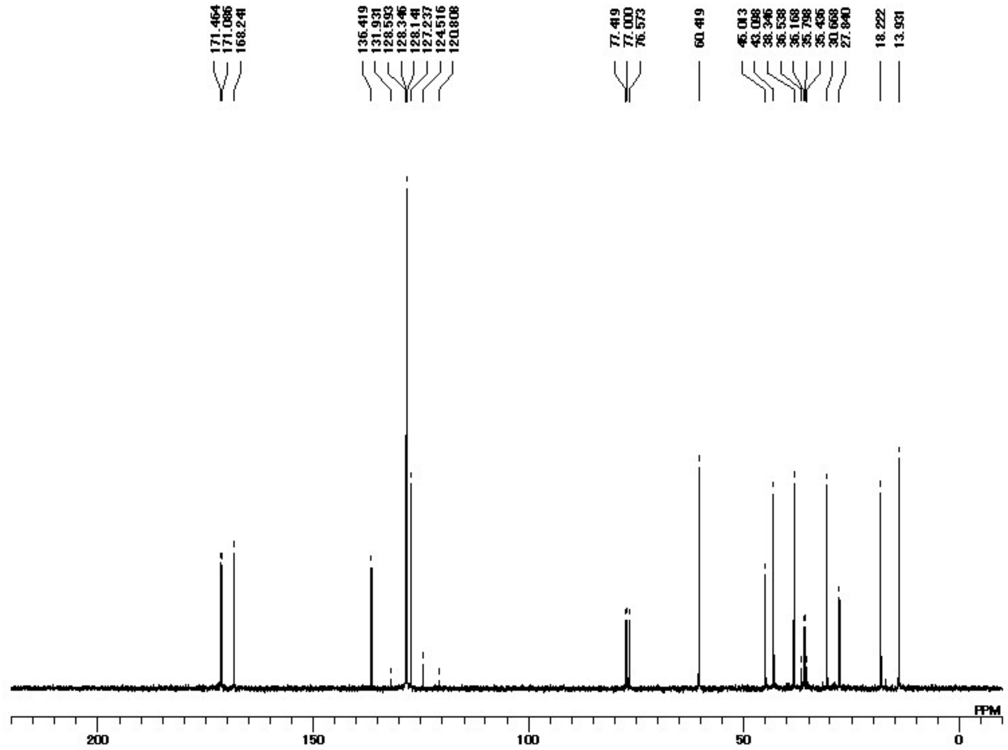

Ethyl 3-{*N*-benzyl-2,6-dioxo-4-(trifluoromethyl)piperidin-3-yl}-3-phenyl-propanoate (3,7-*syn*-7c)

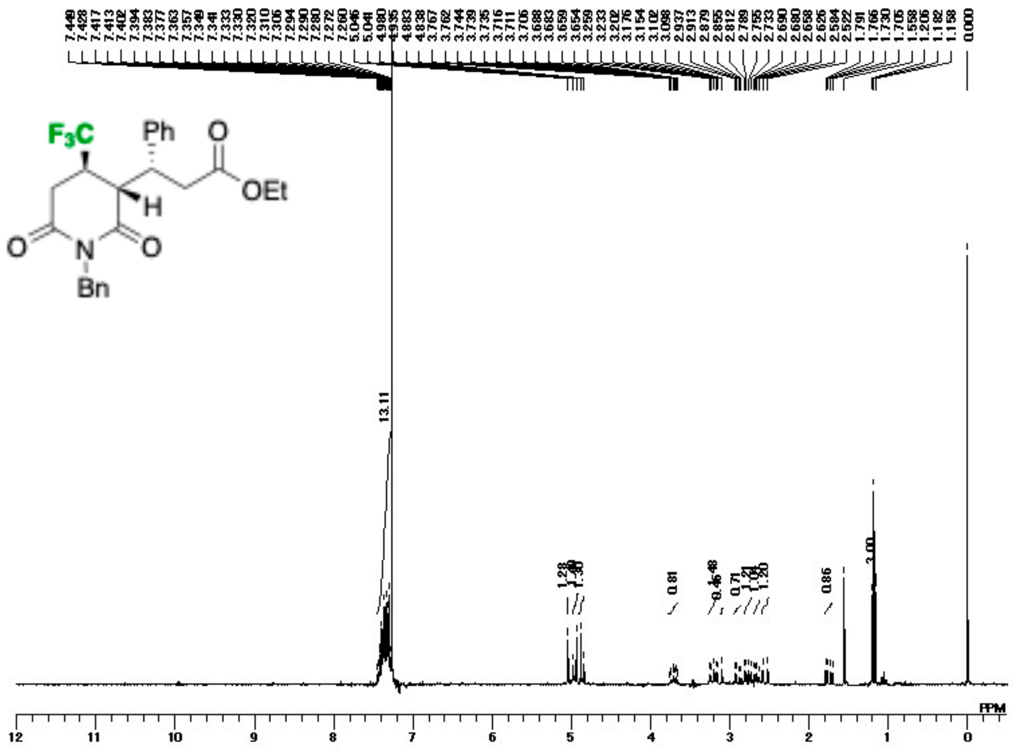

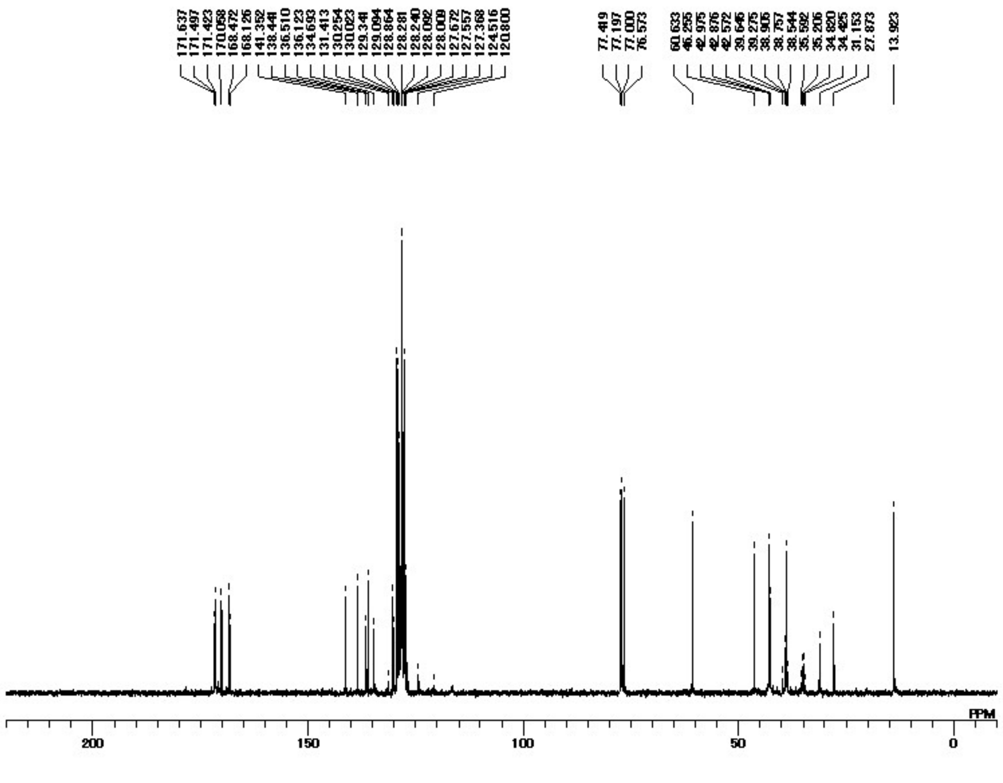

_N_-Benzyl-2-(3-phenylpropan-3-on-1-yl)-3-(trifluoromethyl)glutarimide (3,4-_anti_-7d)

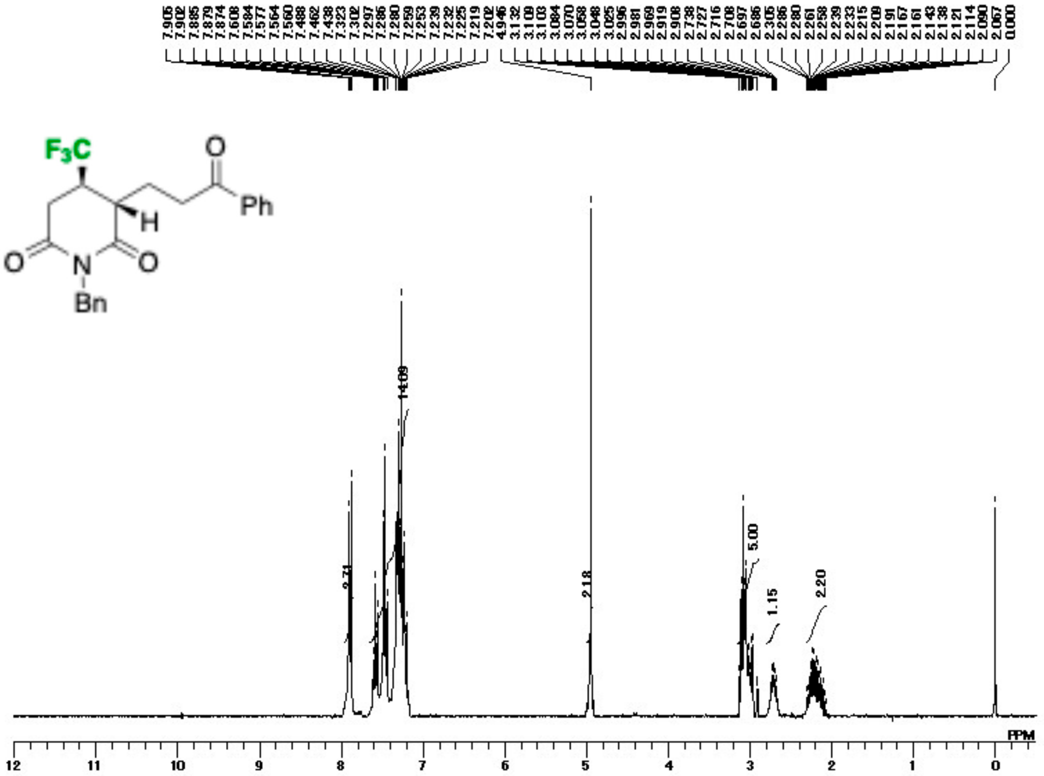

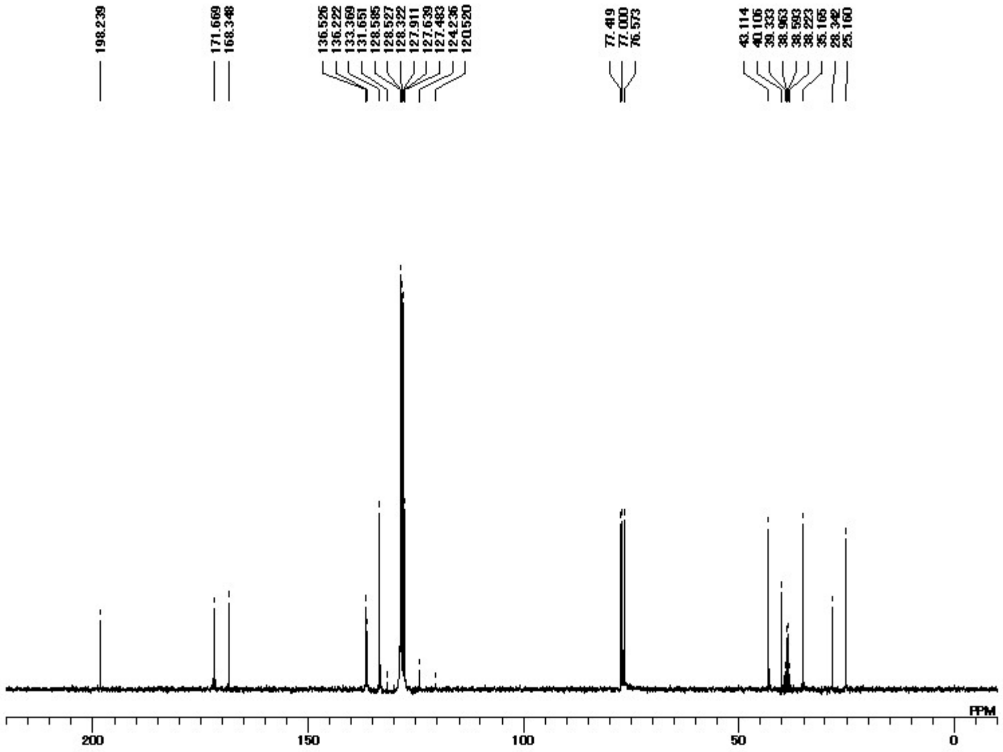

N-Benzyl-2-(cyclohexan-1-on-3-yl)-3-(trifluoromethyl)glutarimide (3,4-*anti*-7g)

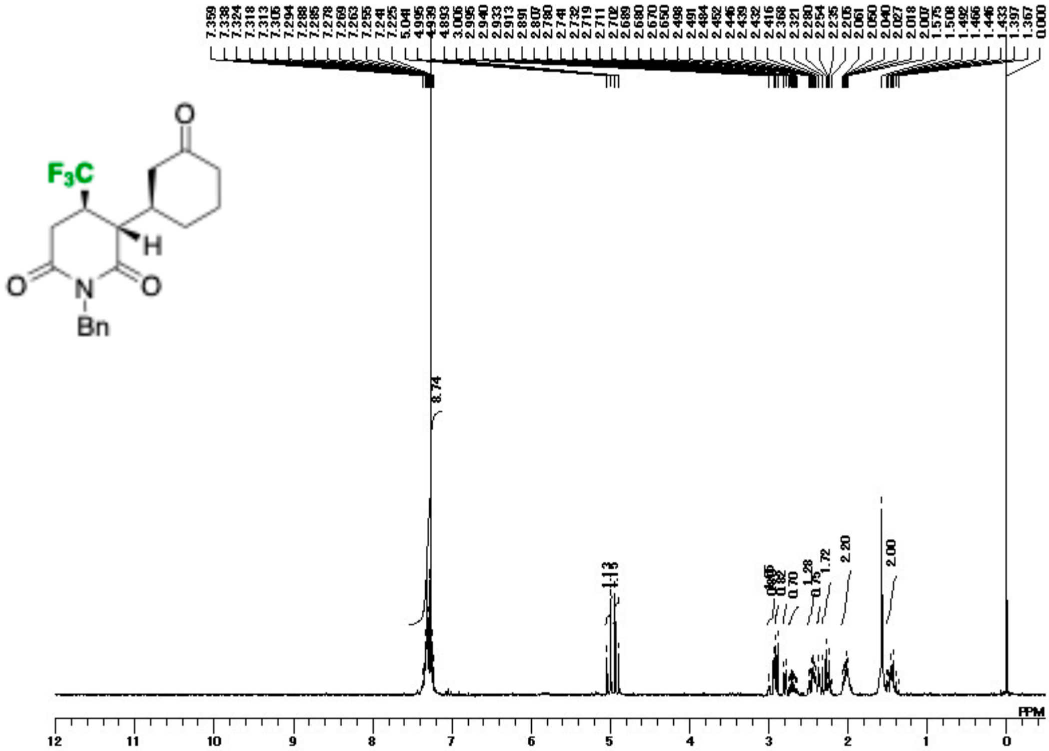

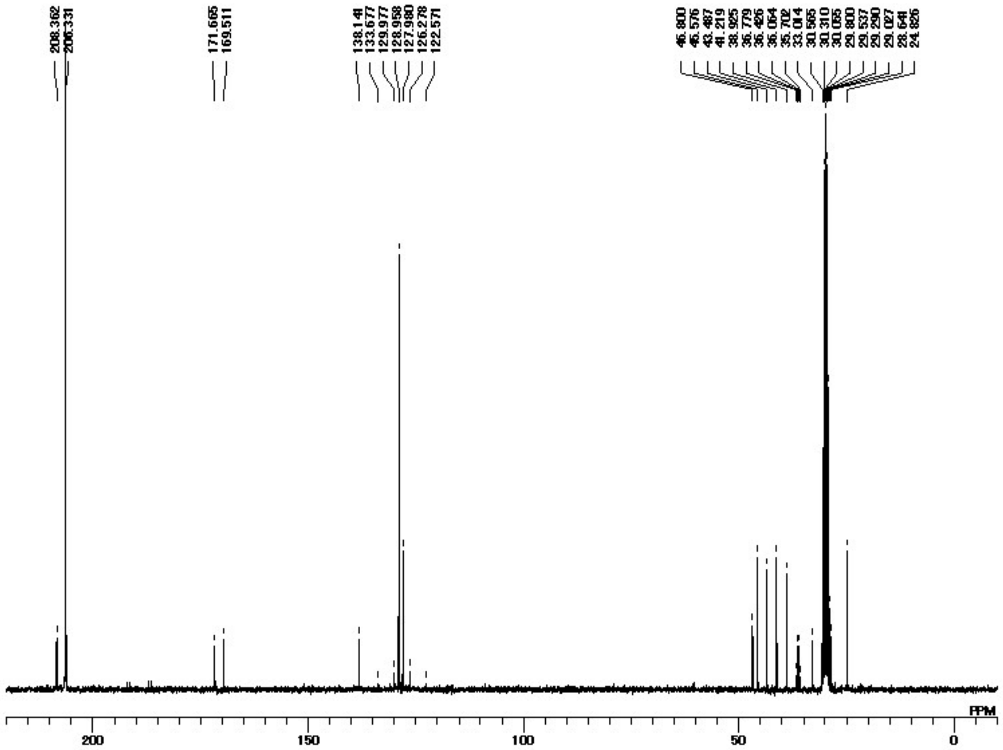

Supplement: Supplementary file 1 [file molecules-29-05129-s001.zip › molecules-3253760-supplementary.pdf]
